# P2X7 Receptor-Dependent Layer-Specific Changes in Neuron-Microglia Reactivity in the Prefrontal Cortex of a Phencyclidine Induced Mouse Model of Schizophrenia

**DOI:** 10.3389/fnmol.2020.566251

**Published:** 2020-11-11

**Authors:** Stefano Calovi, Paula Mut-Arbona, Pál Tod, András Iring, Annette Nicke, Susana Mato, E. Sylvester Vizi, Jan Tønnesen, Beata Sperlagh

**Affiliations:** ^1^Laboratory of Molecular Pharmacology, Institute of Experimental Medicine, Budapest, Hungary; ^2^János Szentágothai Doctoral School, Semmelweis University, Budapest, Hungary; ^3^Walther Straub Institute of Pharmacology and Toxicology, Faculty of Medicine, LMU Munich, Munich, Germany; ^4^Achucarro Basque Center for Neuroscience, Leioa, Spain; ^5^Department of Neuroscience, University of the Basque Country (UPV/EHU), Leioa, Spain; ^6^Centro de Investigación Biomédica en Red Sobre Enfermedades Neurodegenerativas (CIBERNED), Madrid, Spain; ^7^Biocruces Bizkaia, Barakaldo, Spain

**Keywords:** P2X7 receptor, schizophrenia, microglia, phencyclidine, prefrontal cortex

## Abstract

**Background**: It has been consistently reported that the deficiency of the adenosine triphosphate (ATP) sensitive purinergic receptor P2X7 (P2X7R) ameliorates symptoms in animal models of brain diseases.

**Objective**: This study aimed to investigate the role of P2X7R in rodent models of acute and subchronic schizophrenia based on phencyclidine (PCP) delivery in animals lacking or overexpressing P2X7R, and to identify the underlying mechanisms involved.

**Methods**: The psychotomimetic effects of acute i.p. PCP administration in C57Bl/6J wild-type, P2X7R knockout (P2rx7^−/−^) and overexpressing (P2X7-EGFP) young adult mice were quantified. The medial prefrontal cortex (mPFC) of P2rx7^−/−^ and heterozygous P2X7-EGFP acutely treated animals was characterized through immunohistochemical staining. The prefrontal cortices of young adult P2rx7^−/−^ and P2rx7^tg/+^ mice were examined with tritiated dopamine release experiments and the functional properties of the mPFC pyramidal neurons in layer V from P2rx7^−/−^ mice were assessed by patch-clamp recordings. P2rx7^−/−^ animals were subjected to a 7 days subchronic systemic PCP treatment. The animals working memory performance and PFC cytokine levels were assessed.

**Results**: Our data strengthen the hypothesis that P2X7R modulates schizophrenia-like positive and cognitive symptoms in NMDA receptor antagonist models in a receptor expression level-dependent manner. P2X7R expression leads to higher medial PFC susceptibility to PCP-induced circuit hyperactivity. The mPFC of P2X7R knockout animals displayed distinct alterations in the neuronal activation pattern, microglial organization, specifically around hyperactive neurons, and were associated with lower intrinsic excitability of mPFC neurons.

**Conclusions**: P2X7R expression exacerbated PCP-related effects in C57Bl/6J mice. Our findings suggest a pleiotropic role of P2X7R in the mPFC, consistent with the observed behavioral phenotype, regulating basal dopamine concentration, layer-specific neuronal activation, intrinsic excitability of neurons in the mPFC, and the interaction of microglia with hyperactive neurons. Direct measurements of P2X7R activity concerning microglial ramifications and dynamics could help to further elucidate the molecular mechanisms involved.

## Introduction

Schizophrenia (SCZ), which has a prevalence of 0.7% in adults, is a severe psychiatric disorder characterized by abnormalities in thought and cognition (Powchik et al., 1998). The pathology of SCZ is typically recognized by multiple psychotic episodes, along with a progressive worsening of social and cognitive abilities and multifactorial tissue deterioration of key brain areas, such as the prefrontal cortex (PFC; Lesh et al., 2011).

Studies of the molecular events related to SCZ point to a basal dysregulation of the glutamatergic system, which consequently damages the dopaminergic and local circuits, affecting distinct, defined areas of the central nervous system (McCutcheon et al., 2020).

However, the effects and mechanism of SCZ, as well as the consequential detrimental cascade, remain unclear. There is a lack of SCZ treatments that adequately treat cognitive symptoms (Tripathi et al., 2018).

The P2X7 receptor (P2X7R) is an ionotropic purinergic nucleotide receptor that is activated by high extracellular adenosine triphosphate (ATP) concentration and triggers an influx of extracellular calcium (Janks et al., 2018). While it is known that P2X7R is expressed in the brain, the exact cell-type specific localization of the receptors has been the subject of extensive debate (Illes et al., 2017; Teresa Miras-Portugal et al., 2017). Novel findings using the enhanced green fluorescent protein P2X7-EGFP reporter mouse line, which overexpresses the EGFP C-terminally tagged receptor protein, indicates that P2X7R, at least in the mouse brain, is preferentially expressed by microglia and oligodendroglial cells (Kaczmarek-Hajek et al., 2018). In addition to its recognized function as a ligand-dependent ion channel receptor, more than 20 proteins involved in immune functions have been reported to interact with the long intracellular C-terminal tail (Kim et al., 2001; Kopp et al., 2019) of the P2X7R protein.

P2X7R has received considerable interest in recent years due to its widespread involvement in a variety of central nervous system pathologies (Sperlágh and Illes, 2014), including mental health disorders (Bhattacharya, 2018). The first observation that suggested the involvement of P2X7R in psychotic episodes was that receptor deficiency blocked amphetamine-induced hyperactivity in P2X7R deficient mice (Csölle et al., 2013), which observation was further supported by others (Bhattacharya et al., 2013; Lord et al., 2014; Gubert et al., 2016).

More recently, we showed that P2X7R genetic deletion or pharmacological blockade alleviates the acute psychotomimetic effects of phencyclidine (PCP, 2, and 5 mg/kg; Koványi et al., 2016), which is a validated model for SCZ symptomatology (Paasonen et al., 2017). These changes were accompanied by alterations in SCZ-related genes in the PFC (Koványi et al., 2016). However, it is unclear how changes in the activity of P2X7R leads to behavioral alterations.

Extracellular purines, including ATP, adenosine diphosphate (ADP), and adenosine, act as signaling molecules, guiding numerous types of rapid microglial dynamics and interactions in the brain, and modulate long-term physiological cellular functions (Calovi et al., 2019). Among their receptors, P2X7Rs promote microglial proliferation and activation (Bianco et al., 2006; Monif et al., 2010) and the release of proinflammatory cytokines such as interleukin-1β (IL-1β; He et al., 2017).

Activation of P2X7R in the central nervous system as a trigger of microglia-associated neuroinflammation in the prefrontal and hippocampal regions has been recently hypothesized to play an important role in psychiatric disorders (Illes et al., 2020).

PCP is a potent non-competitive N-methyl-D-aspartate receptor (NMDA-R) antagonist that can result in symptoms such as hallucinations, delusions, and disorganized speech, thereby mimicking the episodic phase of psychosis in humans (Bubeníková-Valešová et al., 2008). The class of arylcyclohexylamine anesthetics, such as ketamine and PCP, exerts a complex array of behavioral and neurochemical effects in the brain, which are considered part of the basis of the NMDA-R hypofunction hypothesis of SCZ (Javitt and Zukin, 1991; Castañé et al., 2015). Systemic injection of PCP in rodents results in a psychotomimetic effect, characterized by hyper locomotor activity and stereotypical rotational behavior (Ishmael et al., 1998). Moreover, it is established that acute PCP administration alters the activity of several cortical and subcortical areas, which can be observed by the increased number of c-Fos expressing neurons (Celada et al., 2013; Hervig et al., 2016). In contrast, acute PCP administration in brain slices (Bourne et al., 1983; Wang and Liang, 1998) or local drug delivery in freely moving rats (Suzuki et al., 2002) inhibits most multi-synaptic excitatory neuronal activity.

Interestingly, one of the target areas of PCP-induced enhancement of neuronal activity in rodents is the medial PFC (mPFC; Jodo et al., 2005). The mPFC is intimately involved in cognitive functions, hereunder working and episodic memory, which is disrupted in the PCP model (Arime and Akiyama, 2017).

There are different hypotheses, supported by evidence and not mutually exclusive, to explain the paradoxical effect of systemic vs. local PCP on neuronal circuits. First, the direct preferential inhibition of GABAergic interneurons, predominantly the parvalbumin (PV) axo-axonic cell population, which, by disinhibition of the pyramidal cells, leads to a loss of activity synchronization (Lewis and Gonzalez-Burgos, 2006). Second, indirect activation occurs *via* excitatory firing from the thalamocortical projections of midline nuclei-relay neurons. In this sense, it is supposed that the main site of the PCP inhibitory action is in the reticular nucleus of the thalamus (Celada et al., 2013). The study of thalamocortical projections requires experiments to be conducted *in vivo*. It is also known that acute PCP deeply affects the midbrain dopaminergic system, increasing extracellular levels of dopamine in the frontostriatal region concomitantly with the behavioral psychotomimetic effect (French, 1994; Moghaddam and Adams, 1998). More recently, it has been confirmed that glutamate, noradrenaline, and serotonin rapidly increase in the mPFC upon acute PCP (Kehr et al., 2018).

As mentioned above, PCP-induced neuropathological mechanisms unleash desynchronization and ungoverned activation in most SCZ-related CNS regions (Kargieman et al., 2007), resulting in lower inter-area functional connectivity (Paasonen et al., 2017).

Rodents that undergo repeated PCP treatments experience a complex cumulative effect, which persists during drug withdrawal, leading to a well-documented mimicry of SCZ-negative symptomatology (Jentsch et al., 1998; Castañé et al., 2015; Cadinu et al., 2018). The nature of this long-lasting detrimental effect is still unclear, yet physiological changes are consistently observed in rodent models of NMDA-R antagonists. Deterioration of PV interneurons (Toriumi et al., 2016), network-level abnormalities (Seshadri et al., 2018), and task-related recruitment of neurons (Arime and Akiyama, 2017) are similar to the schizophrenic pathological events in the mPFC of rodents, which corresponds to the dorsolateral frontal cortex in humans. The PFC is not the only area involved in the pathology of PCP models (Uramura et al., 2014).

Interestingly, mild brain inflammation is well documented in SCZ (Müller, 2018), and hampering the microglia proinflammatory shift is suggested to be beneficial in SCZ models (Mattei et al., 2014). Whether neuroinflammation also occurs in the mPFC in PCP SCZ models remains unknown (Zhu et al., 2014).

Therefore, we have aimed to investigate the development of a chronic, unceasing proinflammatory state in the PFC during PCP subchronic treatment, since neuroinflammation might be an interesting, novel pathway through which P2X7R may contribute to the symptomatology of SCZ. However, we have not found evidence that suggested neuroinflammation at any point in the project. Therefore we rejected the potential involvement of chronically activated microglia and neuroinflammation in the P2X7R mediated symptomatology during SCZ.

The present study was designed to study the role of mPFC P2X7 receptors in an acute PCP model for positive symptoms. We have also extended our previous observations to a P2rx7^−/−^ subchronic PCP model, which better reflects the disease phenomenon in humans. Moreover, we tested the heterozygous animals overexpressing P2X7-EGFP (Kaczmarek-Hajek et al., 2018) using the acute treatment model of either a regular or a low dosage of PCP, which better depicts an array of negative symptoms (Paasonen et al., 2017).

## Materials and Methods

### Animals

The behavior, histology, microscopy, cytokine quantification, and ^3^H-Dopamine release experiments were conducted following the principles and procedures outlined in the National Institutes of Health Guide for the Care and Use of Laboratory Animals and were approved by the local Animal Care Committee of the Institute of Experimental Medicine (Budapest, Hungary, ref. No. PEI/001/778-6/2015). The electrophysiology experiments were approved and performed in compliance with National and European regulations (RD1201/2005; 86/609/CEE) following the guidelines of the International Council for the Laboratory Animal Science at the University of the Basque Country UPV/EHU (CEEA 290/2015).

Sixty to ninety days old adult male, drug and test naïve, wild-type (P2rx7^+/+^), and P2X7R knockout (P2rx7^−/−^) mice were housed in a light- (12 h on, 12 h off) and a temperature-controlled room with food and water available *ad libitum*.

Homozygous P2X7R P2rx7^+/+^ mice were bred on the C57Bl/6J background. The P2rx7^−/−^ mice were kindly supplied by Christopher Gabel from Pfizer, Inc. (Groton, CT, USA) or purchased (for electrophysiology experiments, Charles River). The animals contained the DNA construct previously shown to delete the P2X7R (Solle et al., 2001). Offspring of this mouse line was cross-bred with P2rx7^+/+^ mice, and the resulting heterozygotes were used as breeding stock for the F1 generation offspring employed in the behavioral studies. Genomic DNA was isolated from the tails of P2rx7^+/+^ and P2rx7^−/−^ animals, and the genotypes were confirmed by polymerase chain reaction (PCR) analysis.

The overexpressing P2X7-EGFP C57Bl/6J mouse (line 17 in C57Bl6N) was obtained from Annette Nicke (Kaczmarek-Hajek et al., 2018) and bred heterozygously. Mice were raised in grouped cages until the 2 month of life. Male animals were tested using the experiments detailed below.

### Treatments

All treated animals were handled and habituated to the behavioral unit at least 24 h before the first injection. Injections were performed in a treatment room, and behavioral protocols were recorded in a separate experimental room.

Acute PCP treatment consisted of a single i.p. injection (volume of 10 ml/kg) of the vehicle (0.9% NaCl sterile) or PCP at two dosages, referred to as PCP and low-dose PCP (PCP-HCl 10 mg/kg and low dose 2 mg/kg, Sigma–Aldrich Kft, Budapest, Hungary) freshly dissolved in the vehicle. Ten animals per genotype (P2rx7^−/−^ and P2rx7^+/+^) and 12 animals per genotype (P2rx7^tg/+^ and P2rx7^+/+^) were deeply anesthetized (Nembutal 100 mg/kg, Sigma–Aldrich Kft, Budapest, 10 ml/kg injection volume) 180 min after receiving PCP treatment, and transcardially perfused 10 or 20 min afterward (Figures 2A, 9A). For the low-dose PCP experiment, 18 animals homozygous for P2X7R and heterozygous for P2X7-EGFP protein overexpression (Kaczmarek-Hajek et al., 2018), referred to as P2rx7^tg/+^, were randomized into a vehicle and low-dose PCP groups. Animals were habituated 30 min before the experimental environment, received the treatment, and were placed back in the house cage for 45 min. Then, they were tested in pairs for 10 min in a circular open field (8A). Subsequently, they were deeply anesthetized (Nembutal) and transcardially perfused.

**Figure 1:**
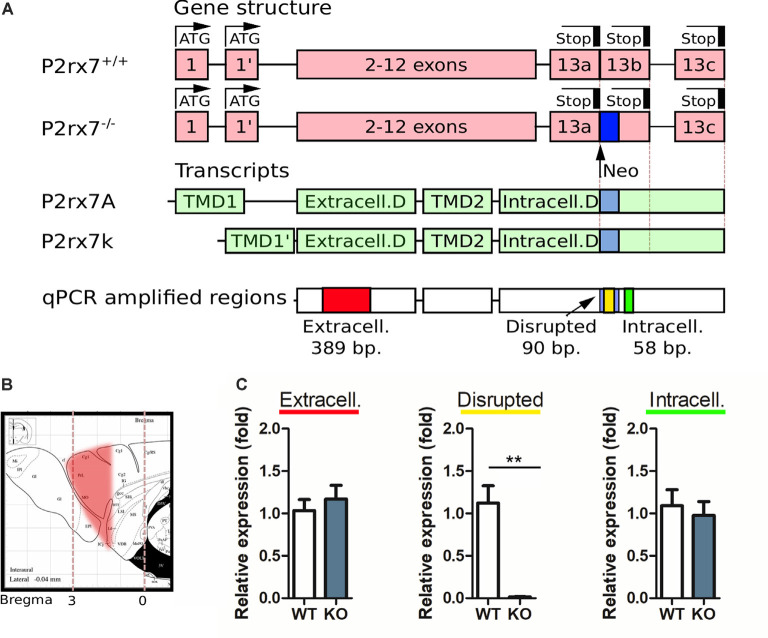
Purinergic receptor P2X7 (P2X7R) expression analysis in the prefrontal cortex with Real-time quantitative polymerase chain reaction (RT-qPCR). **(A)** Schematic representation of the P2rx7 gene in P2rx7^+/+^ and P2rx7^−/−^ mouse lines, the principal splicing variants found in C57bl/6 line, and the amplified regions in the qPCR. **(B)** Schematic representation of the prefrontal cortex (PFC) cut, subsequently analyzed. Paxinos ATLAS, sagittal. **(C)** Purinergic receptor P2X7 (P2X7R) expression analysis in P2rx7^+/+^ and P2rx7^−/−^ mouse (*N* = 5, both groups) prefrontal cortex by real-time qPCR. Relative expressions were given as fold changes normalized to GAPDH. Shown is the mean ± SEM. Statistical analysis was performed with unpaired Student’s *t*-test, *p*-value **< 0.01 **(C)**.

**Figure 2:**
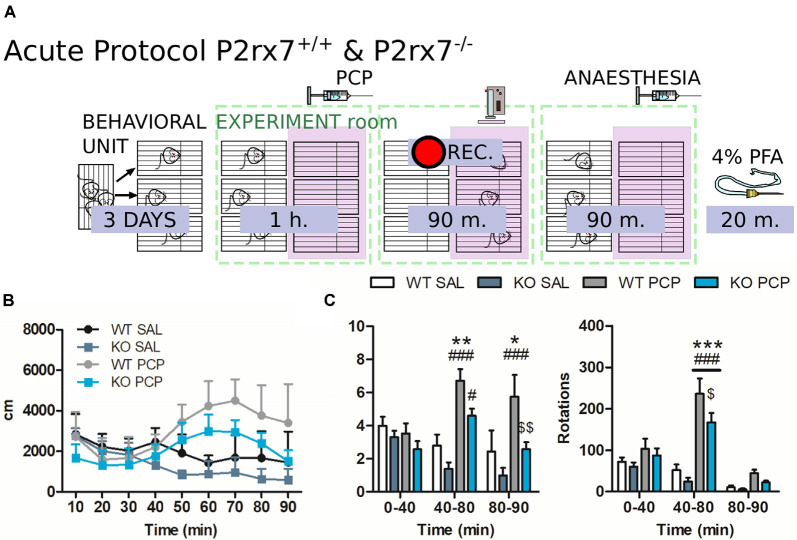
P2rx7^−/−^ mice are less susceptible to the acute phencyclidine (PCP) psychotomimetic effect. **(A)** Schematic representation of the behavioral protocol for acute treatment. The vehicle or PCP 10 mg/kg i.p. injection is represented by the PCP syringe, the pink panel represents the IR backlight, and the pink rectangle in front of the camera represents the infrared (IR) filter. **(B)** Distance moved by the animals after the injection, in 10 min time bins. **(C)** Average velocity (left) and numbers of circle rotations (right) averaged for the first and second 40 min, plus the remaining recorded time. The PCP psychotomimetic effect was evident 40 min after the PCP injection. *N* = 4 (WT Sal), *N* = 5 (KO PCP), and *N* = 6 (WT PCP). Shown is mean ± SEM. Statistical analysis was performed with two-way analysis of variance (ANOVA) followed by Bonferroni’s *post hoc* test. Scoring: +; ++; +++; mean *p*-values < 0.05; 0.01; 0.001. Symbols: * vs. WT SAL; ^#^ vs. KO SAL; ^$^ vs. WT PCP.

**Figure 3:**
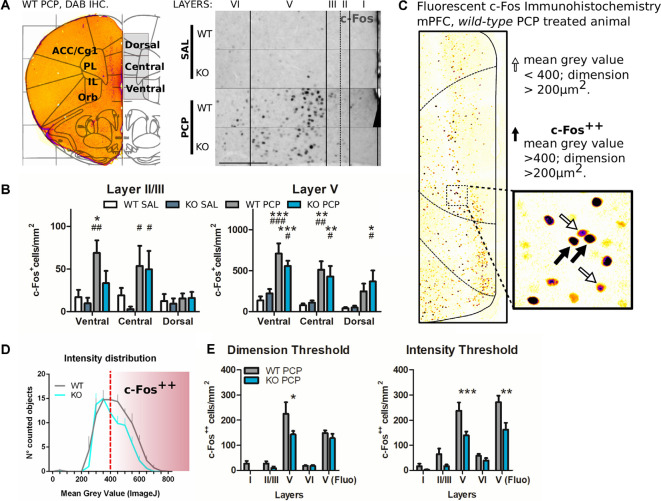
The reduced PCP related behavioral outcome in P2rx7^−/−^ is correlated with lower PCP-driven hyperactivation of the medial PFC mPFC neurons. **(A)** Representative c-Fos/DAB immunostaining of the coronal PFC from a P2rx7^+/+^ PCP treated mouse overlapped with the Paxinos ATLAS (Bregma +2.00; Franklin and Paxinos, 1997). The left hemisphere represents the field of view of the pictures quantified (left). Representative immunostaining from projected images used for automated c-Fos nuclei counting. Layer width ranges: *I* = 120–140 μm; II/III = 80 μm; *V* = 320–350 μm; VI = minimum 280 μm. Scale bar: 200 μm (right). **(B)** Quantification of c-Fos positive nuclei in layers II/III and V of the mPFC. **(C)** Representative picture of the coronal PFC immunostaining (WT PCP) with a different c-Fos antibody (SYSY Cfos-226 004 guinea pig/fluorescent Jackson IR secondary). In the V-centered insert layer, the white arrows indicate c-Fos positive nuclei (mean gray value <400, dimension >200 μm^2^), while the black arrows indicate strongly immunostained nuclei (mean gray value >400; dimension >200 μm^2^), referred to as c-Fos^++^. The length of the insert square side is 165 μm. **(D)** Averaged histogram of the mean gray value for the automatically detected c-Fos positive nuclei in layer V. PCP-treated P2rx7^−/−^ and P2rx7^+/+^ prelimbic and infralimbic areas were quantified as the central and dorsal portions of the ventral mPFC pictures. The red dashed line represents the threshold (400 units of mean gray value, FIJI ImageJ), which were considered strongly activated neurons c-Fos^++^. **(E)** Quantification of prelimbic and infralimbic content of strongly activated c-Fos^++^ nuclei, by dimension (left) and intensity (right) threshold. Automatic counting with FIJI ImageJ in each medial PFC layer for DAB immunostaining, and in the isolated layer V for fluorescent immunostaining. *N* = 3 (Sal), *N* = 4 (PCP). Shown is mean ± SEM. Statistical analysis was performed with two-way ANOVA followed by Bonferroni’s *posthoc* test. Scoring: +; ++; +++; mean *p*-values < 0.05; 0.01; 0.001. Symbols: **(B)** * vs. WT SAL; ^#^ vs. KO SAL; ^$^ vs. WT PCP; **(E)** * vs. WT PCP.

**Figure 4:**
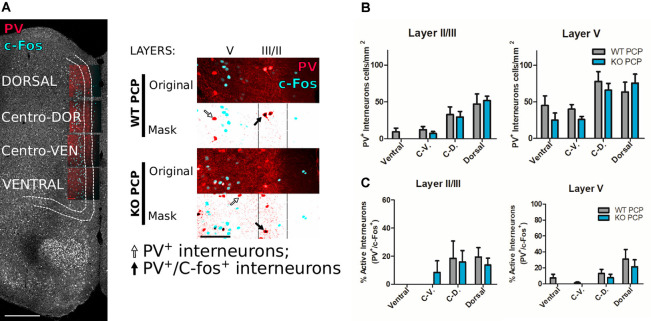
Medial prefrontal parvalbumin (PV) interneurons do not differ in terms of numbers and levels of activity in PCP-treated P2rx7^−/−^ and P2rx7^+/+^ animals. **(A)** Examples of double immunostaining for c-Fos and PV. Reconstructed mPFC from a P2rx7^+/+^ phencyclidine (PCP) treated (WT PCP) mouse. Scale bar, 500 μm (left). Representative images of dorsal mPFC double immunostaining (right). The original pictures are the stacks maximal projections while masks are the correspondent threshold images, combined to facilitate the manual counting of PV^+^ interneurons (black arrow) and double PV^+^ and c-Fos^+^ (white arrow) interneurons in the II/III (80 μm) and V (320–350 μm) layers. Scale bar 100 μm. **(B)** Results of manual counting of PV^+^ interneurons concentrations in mPFC of PCP-treated animals. **(C)** Results of the manual counting of double-positive PV^+^ and c-Fos^+^ interneurons expressed as the percentage of the counted PV^+^ interneurons in the mPFC. *N* = 4 (WT PCP), *N* = 3 (KO PCP). Shown is mean ± SEM. Statistical analysis was performed with two-way ANOVA followed by Bonferroni’s *posthoc* test. Scoring: no significant differences, *p* value > 0.05.

**Figure 5:**
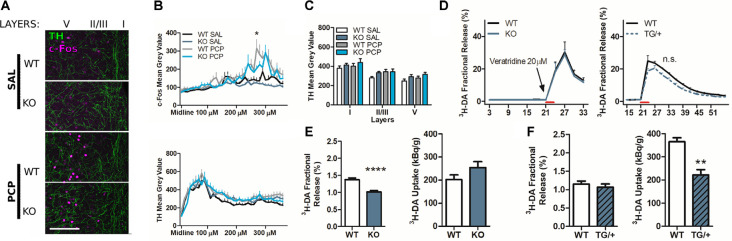
The P2rx7^−/−^ prefrontal cortex displays a lower dopamine release, while P2rx7 overexpression leads to lower dopamine uptake compared to the respective wild-type (WT) prefrontal cortex. **(A)** Example of P2rx7^−/−^ and P2rx7^+/+^ mPFC double immunostaining for c-Fos and the dopamine- and noradrenaline-synthesizing fiber marker tyrosine hydroxylase (TH). Z-stack maximal projections. Scale bar 100 μm. **(B)** Longitudinal quantification, with FIJI ImageJ, of the c-Fos (up) and TH (down) immunoreactivity. The profiles of the average signal along the mPFC medial-distal axis show that layer I, where the peak expression of TH fibers is detected, does not correspond with the peak of c-Fos activated neurons (layer V). **(C)** Quantification of TH expression. The profile intensities were grouped by layers. *N* = 2; 6 pictures per animal. Shown is mean ± SEM. Statistical analysis was performed with two-way ANOVA followed by Bonferroni’s *posthoc* test. Scoring: +; means *p*-value < 0.05. Symbols: * WT PCP vs. KO PCP at 270 μm and 280 μm. **(D)** Graph displays stimulated fractional release from PFC slices induced by 20 μM veratridine perfusion. *N* = 28 (WT); *N* = 11 (KO), left; *N* = 4 (WT); *N* = 4 (TG/+), right. Shown is mean ± SEM. Statistical analysis was performed with two-way ANOVA followed by Bonferroni’s *posthoc* test. Scoring: no significant differences, *p*-value > 0.05. **(E)** Results of the P2rx7^−/−^ [^3^H]-DA release experiments. The quantification of dopamine release and uptake confirm the previously published data (Koványi et al., 2016). *N* = 14 (WT); *N* = 8 (KO). **(F)** Results of the P2rx7^tg/+^ [^3^H]-DA release experiments. The quantification of dopamine release and uptake suggests that the newly tested P2X7R-EGFP line display lower DA uptake concerning the wild type cortex; *N* = 4 (WT) *N* = 4 (TG/+). Shown is mean ± SEM. Statistical analysis was performed with unpaired Student’s *t*-test with Welch corrections. Scoring: ++; ++++; means *p*-values < 0.01; 0.0001. Symbols: **(E,F)** * vs. WT.

**Figure 6:**
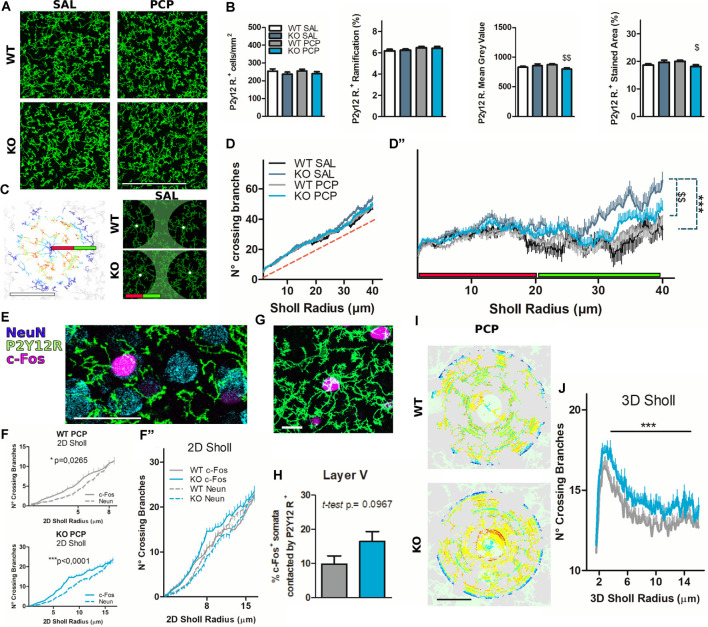
Microglial cells organize their processes around hyperactivated neurons, and their ramification is partly dependent on P2X7 receptor (P2X7R) expression. **(A)** Representative images of mPFC layer V immunostaining for the microglial membrane marker P2Y12R. Scale Bar, 100 μm. **(B)** Bar diagrams quantify P2Y12R positive cell properties characterization. Cell count was manually performed (left). The general ramification was quantified with FIJI ImageJ as the percentage of the stained area from the skeleton stacks (left center). The signal intensity was assessed with FIJI ImageJ as the mean gray value (center-right) and percentage of threshold stained area (right). *N* = 3 (Saline, Sal), *N* = 4 (phencyclidine, PCP). **(C)** Example of 2D Sholl analysis of the mPFC layer V non-isolated microglia (WT SAL), after processing the skeleton of the maximal stack projection, all performed in FIJI ImageJ. The first 20 μm of the Sholl radius is likely to be occupied by a single-cell harborization (green line); from 20 μm to 40 μm of Sholl radius (inter-cellular territory) are present ramifications of several cells (red line). Scale bar, 40 μm (left). Representative images of the difference in inter-cellular territory ramification from vehicle-treated P2rx7^+/+^ and P2rx7^−/−^ microglia. The green-colored areas were approximately 20 μm from the microglia nuclei signed with white spots (right). **(D)** Quantification of the 2D Sholl analysis is represented as the number of crossing branches per radius value of non-isolated microglial cells. The red dashed line is the identity function *y* = *x*. **(D″)** The same graph presented in **(D)**, but the y-axis has changed with the identity function *y* = *x* for better visualization. *N* = 3 (Sal), *N* = 4 (PCP); 2D cell-center numbers analyzed: *n* = 38 (WT SAL), *n* = 48 (KO SAL), *n* = 60 (WT PCP), *n* = 59 (KO PCP). Shown is mean ± SEM. Statistical analysis was performed Mann–Whitney *U-test* over the curves between 0 and 20 μm (not significant) and 20–40 μm. **(E)** Example of triple immunostaining of the mPFC layer V for microglia (P2Y12R), neuronal nuclei (NeuN), and activated neuronal nuclei. Scale Bar 30 μm. **(F)** Quantification of the 2D Sholl profiles of microglial branches surrounding PCP-activated (continuous line) or inactive (dashed line) neuronal nuclei. Wild-type PCP-treated animals, in prelimbic and infralimbic layer V areas, displayed preferential microglial contact in the 8 μm radius, still targeting c-Fos^+^ activated neurons after 180 min from the PCP-induced psychotomimetic effect plus 20 min after Nembutal anesthesia. The preferential microglial contact towards activated neurons was even larger, in terms of the radius (15 μm) in P2rx7^−/−^ animals. *N* = 4; 2D cell-center numbers analyzed: *n* = 52 (WT NeuN), *n* = 51 (KO NeuN), *n* = 34 (WT c-Fos), *n* = 36 (KO c-Fos). Shown is mean ± SEM. Statistical analysis was performed with two-way ANOVA followed by Bonferroni’s *posthoc* test, exact *p*-values are displayed on graphs. **(F″)** Values presented in *F* grouped in a single chart. In the 8 μm Sholl radius, c-Fos vs. NeuN in two-way ANOVA Bonferroni post hoc test, for both genotypes, return *p-values* > 0.05. **(G)** Example of mPFC layer V double immunostaining representing P2Y12R positive microglia in which the cell body is in physical contact with an activated c-Fos^+^ neuronal nucleus. Scale bar 15 μm. **(H)** Quantification of the manually counted cell-body contacts between the microglia and activated neurons expressed as a percentage of counted c-Fos^+^ nuclei. *N* = 4. Shown is mean ± SEM. Statistical analysis was performed with unpaired Student’s *t*-test, the exact *p*-value shown on the graph, not significant. **(I)** Example of c-Fos^+^ nuclei centered 3D Sholl analysis of P2Y12R^+^ microglial cell membrane in the mPFC layer V from PCP-reacted P2rx7^+/+^ and P2rx7^−/−^ mice. Scale bar 15 μm. **(J)** Results from the quantification of the 3D Sholl analysis. *N* = 3; 3D cell-center numbers: *n* = 52 (WT PCP); *n* = 45 (KO PCP). Shown is mean ± SEM. Statistical analysis was performed with Mann–Whitney *U*-test. Scoring: +; ++; +++; means *p*-values < 0.05; 0.01; 0.001. Symbols: **(B)** ^$^ vs. WT PCP; **(D”)** ^#^ vs. KO SAL; ^$^ vs. WT PCP; **(F)** * c-Fos vs. NeuN; **(J)** * KO PCP vs. WT PCP.

**Figure 7:**
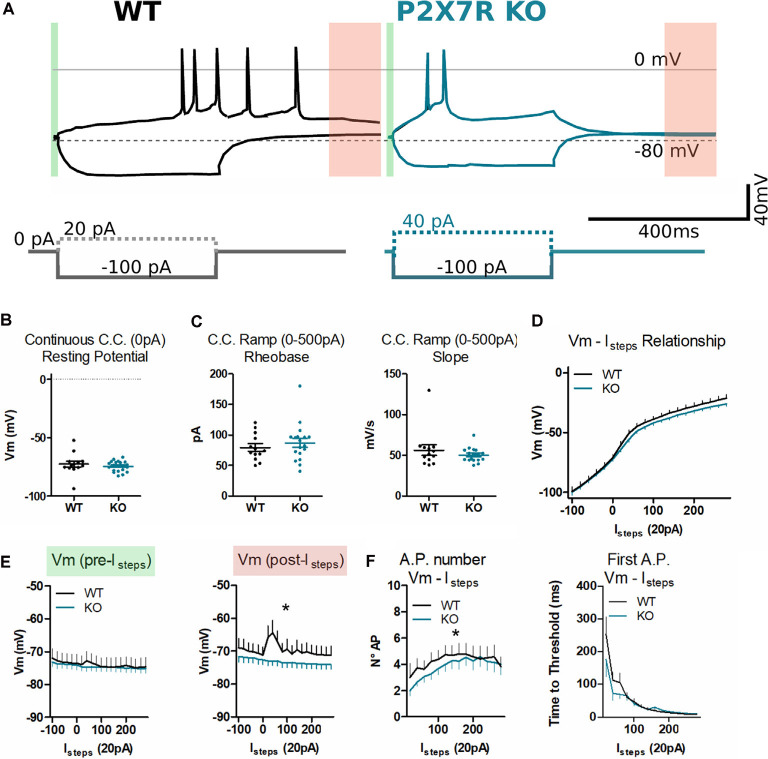
P2rx7^−/−^ mPFC layer V neurons display similar depolarization but faster re-polarization after firing action potentials. **(A)** Example of traces from P2rx7^+/+^ and P2rx7^−/−^ mPFC layer V pyramidal neurons during the whole-cell patch-clamp current step injection (Istep) protocol. Recording of the 400 ms hyperpolarizing step (−100 pA) and the first step triggering action potential firing (+20 pA for P2rx7^+/+^, left; 40 pA for P2rx7^−/−^, right) have been reported. **(B)** The voltage of membrane in the current-clamp configuration. Holding current at 0 pA for 10 s. **(C)** Analysis of the ramp current injection (0–500 pA) over 1 s. Column analysis **(B,C)**, each point represents an individual neuron. *N* = 8 P2rx7^+/+^ (13 neurons); *N* = 6 P2rx7^−/−^ (21 neurons). Shown is mean ± SEM. Statistical analysis was performed with unpaired Student’s *t*-test. Scoring: no significant differences, *p*-value > 0.05. **(D)** Current clamp step protocol (Istep) IV relationship. Istep minimum of −100 pA, Istep maximum of +280 pA, Istep size of 20 pA, Istep time of 400 ms, and sweep frequency of 1 Hz. Averages ± SEM voltage over 400 ms Istep. **(E)** Membrane voltage before and after Istep in the current clamp at 0 mV. Average voltage over 30 ms before Istep [A green, Vm (pre-Istep), left], and over 100 ms [A red, 300–400 ms after Istep, Vm (post-Istep), right]. **(F)** Count of action potentials fired during the 400 ms Istep (left) and plot of the time to the threshold of the first action potential. *N* = 8 P2rx7^+/+^ (12 neurons); *N* = 6 P2rx7^−/−^ (19 neurons). Shown is mean ± SEM. Statistical analysis was performed with Mann–Whitney *U*-test. Scoring: *indicates *p*-value < 0.05.

**Figure 8:**
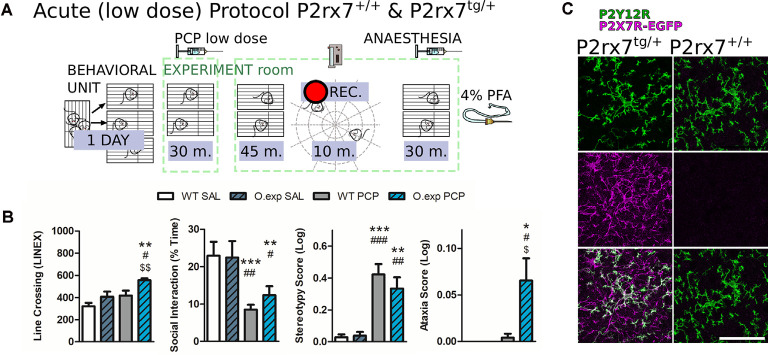
P2rx7^tg/+^ mice are more susceptible to the acute low dose of phencyclidine (PCP) psychotomimetic effect. **(A)** Schematic representation of the behavioral protocol for acute treatment. The vehicle or low dose PCP 2 mg/kg i.p. injection is represented by the PCP syringe. Animals were tested 45 min after receiving the treatment. **(B)** Quantification of different aspects of the PCP-induced behavior in the coupled open-field test. Locomotor activity (left), time spent performing social behavior (left-center), and scores for stereotypical behavior (center right) and ataxia (right) were quantified and analyzed. *N* = 10 (WT SAL), *N* = 12 (O.exp SAL), *N* = 6 (WT PCP), *N* = 12 (O.exp PCP). Shown is mean ± SEM. Statistical analysis was performed with one-way ANOVA followed by Dunn’s comparison *posthoc* test (left and left-center); and Kruskal-Wallis test (center-right and right). Scoring: +; ++; +++; mean *p*-values < 0.05; 0.01; 0.001. Symbols: * vs. WT SAL; ^#^ vs. O.exp SAL; ^$^ vs. WT PCP. **(C)** Example of the P2rx7^tg/+^ layer V mPFC double immunostaining for P2Y12R (green) and GFP, labeling the P2X7R-GFP protein (violet). Scale bar 50 μm.

**Figure 9:**
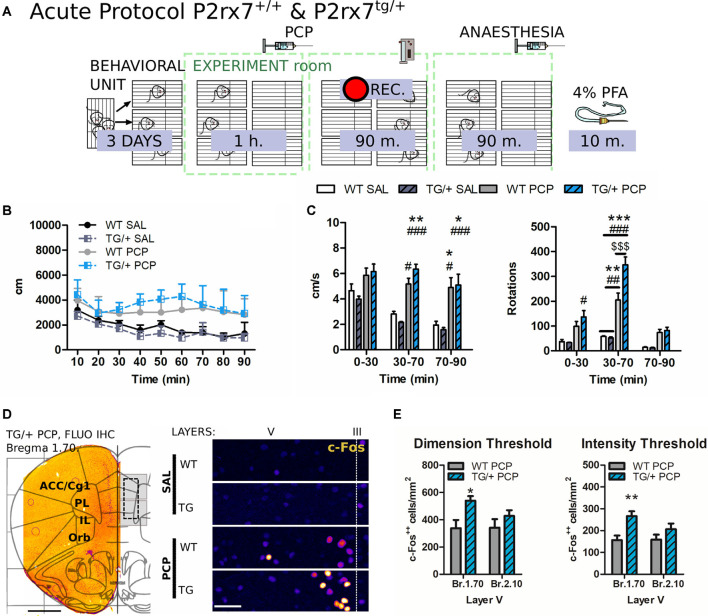
P2rx7^tg/+^ mice display exacerbated acute phencyclidine (PCP) psychotomimetic effect correlated with a higher PCP-driven hyperactivation of the mPFC neurons. **(A)** Schematic representation of the behavioral protocol for acute treatment. The vehicle or PCP 10 mg/kg i.p. injection is represented by the PCP syringe. **(B)** Distance moved by the animals after the injection, in 10 min time bins. **(C)** Average velocity (left) and numbers of circle rotations (right) averaged for the first 30 and second 40 min, plus the remaining recorded time. The PCP psychotomimetic effect was evident 30 min after the PCP injection. *N* = 3 (WT and TG/+ Sal), *N* = 9 (TG/+ PCP), and *N* = 9 (WT PCP). Shown is mean ± SEM. Statistical analysis was performed with two-way ANOVA followed by Bonferroni’s *post hoc* test. **(D)** Representative c-Fos fluorescent immunostaining of the coronal prefrontal cortex (PFC) from a P2rx7^tg/+^ PCP treated mouse overlapped with the Paxinos ATLAS (Bregma +2.00). The left hemisphere represents the field of view of the pictures quantified. Scale bar: 1 mm (left). Representative immunostaining from 5 μm projected images used for automated c-Fos nuclei counting. Layer V width range *V* = 280–350 μm. Scale bar: 50 μm (right). **(E)** Quantification of prelimbic and infralimbic content of strongly activated c-Fos^++^ nuclei, by dimension (left) and intensity (right) threshold. Automatic counting with FIJI ImageJ in the isolated layer V for fluorescent immunostaining. Shown is mean ± SEM. Statistical analysis was performed with unpaired Student’s *t*-test. Scoring: +; ++; +++; mean *p*-values < 0.05; 0.01; 0.001. Symbols: **(C)** * vs. WT SAL; ^#^ vs. TG/+ SAL; ^$^ vs. WT PCP; **(E)** * WT PCP vs. TG/+ PCP.

**Figure 10:**
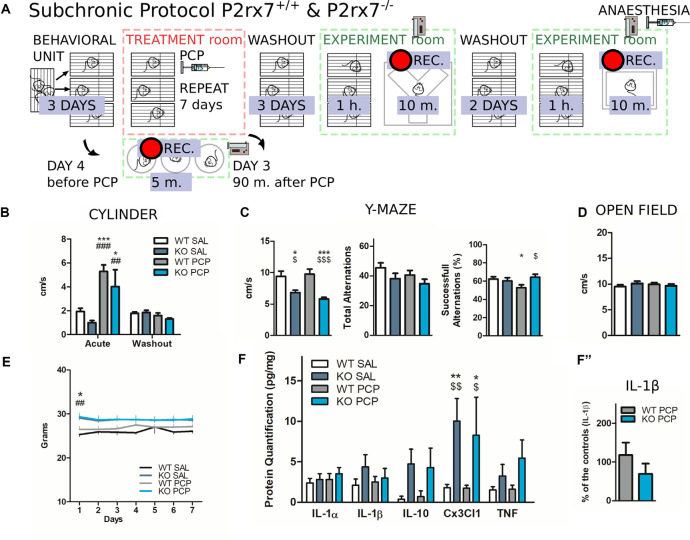
P2rx7 gene-deficient animals show reduced deficit in working memory after PCP subchronic treatment independently of prefrontal neuroinflammation. **(A)** Schematic representation of the behavioral protocol for the subchronic treatment. The vehicle or PCP 10 mg/kg i.p. injections were repeated for seven consecutive days in a treatment room (red-dashed square). Animals were tested during and after the treatment washout a separated experimental room (green-dashed square). **(B)** Velocity inside a glass cylinder during a 5-min-long test. “Acute” refers to the test performed 90 min after the third PCP/Saline injection; (third day); “Washout” refers to the test performed 20 h after the third PCP/Saline injection. *N* = 4. Shown is mean ± SEM. Statistical analysis was performed with two-way ANOVA followed by Bonferroni’s *post hoc* test. **(C)** Results of the Y-maze spontaneous alternation test performed the third day after withdrawal from the subchronic treatment. The animals were placed in the arena and recorded for 10 min. Semi-automated analysis with EthoVision^®^ XT. *N* = 8, Shown is mean ± SEM. Statistical analysis was performed with two-way ANOVA followed by Bonferroni’s *posthoc* test (left, center); unpaired Student’s *t*-test (right, successful alternations). **(D)** Average velocity over 10 min open field test. **(E)** Weight of the animals during the PCP subchronic treatment. *N* = 8, Shown is mean ± SEM. Statistical analysis was performed with two-way ANOVA followed by Bonferroni’s *post hoc* test. **(F)** Interleukins quantification with bead-array FACS analysis from prefrontal cortices of P2rx7^−/−^ and P2rx7^+/+^ vehicle or PCP subchronically treated mice. Brain extraction 15 days after the first injection. **(F″)** Effect of PCP subchronic treatment on the level of IL-1β protein expressed as a percentage of the control. *N* = 8 (Sal), *N* = 12 (PCP), Shown is mean ± SEM. Statistical analysis was performed with two-way ANOVA followed by Bonferroni’s *posthoc* test. Scoring: +; ++; +++; mean *p*-values < 0.05; 0.01; 0.001. Symbols: * vs. WT SAL; ^#^ vs. KO SAL; ^$^ vs. WT PCP.

Subchronic treatment consisted of the daily vehicle or PCP injections for seven consecutive days (Zain et al., 2018). A cohort of 26 P2rx7^+/+^ and 30 P2rx7^−/−^ animals was subchronically treated, followed by a withdrawal period of 7 days before sacrifice (Figure 10A). Four independent treatments with a maximum of eight animals per genotype were conducted. The animals were decapitated after deep anesthesia (Nembutal).

### Real-Time qPCR

To measure the expression of the P2X7 receptor in the prefrontal cortex (PFC), five wild type and knockout mice were sacrificed. The PFC (cut at Bregma 1.20) was snap-frozen in liquid nitrogen and kept at −80°C.

Total RNA isolation was performed using TRI Reagent^®^ (Sigma–Aldrich, St. Louis, MO, USA) according to the manufacturer’s protocol. In brief, chloroform was used for phase separation, the supernatants were aspirated and mixed with propane-2-ol (Sigma–Aldrich, St. Louis, MO, USA) for RNA precipitation. RNA pellets were washed with 70% ethyl alcohol and dissolved in RNase-free water. RNA concentrations were measured using Nanodrop 2000c Spectrophotometer (Thermo Fisher Scientific, Wilmington, DE, USA). The RNA integrity was verified by electrophoretic separation on 1% agarose gel. Reverse transcription from 1 μg RNA into cDNA was performed using the High-Capacity cDNA Archive Kit (Applied Biosystem, Foster City, CA, USA) according to the manufacturer’s protocol. Based on the previously published primer sequences (Sánchez-Nogueiro et al., 2005), the following three regions of the P2X7 receptor were measured by Real-time quantitative PCR (RT-qPCR): (1) the extracellular region; (2) the intracellular region; and (3) the absent region in the knockout mice (disrupted). Primer sequences used to amplify the disrupted region was 5′-TGCATCACCACCTCCAAGCTCTTCCAT-3′ (forward primer) and 5′-CACCAGCAAGGGATCCTGGTAAAGC-3′ (reverse primer); to amplify the extracellular part 5′-GCACGAATTATGGCACCGTC-3′ (forward primer) and 5’-ACACCTGCCAGTCTGGATTCCT-3′ (reverse primer); and to amplify the intracellular part 5′-AGGATCCGGAAGGAGTT-3′ (forward primer) and 5′-TAGGGATACTTGAAGCCACT-3′ (reverse primer) was used. The housekeeping gene, GAPDH, was used for normalization to account for intra-well variability [5′-TTCACCACCATGGAGAGGGC-3′ (forward primer) and 5′-GGCATGGACTGTGGTCATGA-3′ (reverse primer)]. The RT-qPCR reactions were performed using the SensiFast SYBR Green No-Rox kit (Bioline Reagents Limited, London, UK) according to the manufacturer’s protocol in 10 μl total volume. The RT-qPCR products of the P2X7 receptor were visualized by electrophoretic separation on 1% agarose gel (data not shown).

### Behavioral Studies

#### Acute Treatments

P2rx7^+/+^, P2rx7^−/−^ and P2rx7^tg/+^ post-natal day 71–81 male mice were housed in individual cages for 3 days in the behavioral unit, and randomly assigned to the two treatment groups: six received acute PCP treatment and four received vehicle treatment for the wild type vs. knockout experiment, while nine received acute PCP treatment and three received vehicle treatment for the wild type vs. P2X7R overexpressing line experiment. The experiments were repeated independently two and three times for genotype tested, with a maximum of 10 animals tested at a time. In the wild type vs. knockout experiments, the mice within their home cages were placed in the experimental room at dim light for 1 h before each behavioral protocol was performed (Figure 2A). Following this habituation period, the animals were individually transported to the treatment room and injected according to their weight. Once treated, each animal was returned to the experimental room in a clean home-cage positioned over an infrared backlight platform (IR, 850 nm). Each animal left the experimental room to receive treatment for a maximum of 3 min. A fixed camera (Basler aca1300-60gc GigE camera—computer H3Z4512 CS-IR 4.5–12.5 mm F1.2 lens) equipped with an IR filter (Heliopan 35.5 RG850) was mounted on the ceiling of the experimental room. This IR system-generated high-contrast movies that were devoid of reflected-light artifacts, which are common with plexiglass arenas under dim light conditions. In the wild type vs. P2X7R overexpressing line experiments, the protocol was performed in normal light conditions. The animals were recorded continuously for 90 min after the injections. After the recording, the animals were placed back in their home cages for additional 90 min.

For the low-dose PCP study, P2rx7^+/+^ and P2rx7^tg/+^ animals were housed in individual cages in the behavioral unit 24 h before receiving the treatment. For each genotype, eight and six animals were treated with vehicle, and 10 and 12 were subjected to low-dose PCP, respectively. The experiment was independently repeated three times. Animals were habituated to the experimental room for 30 min and were transferred for injection in pairs to the treatment room. Forty-five minutes later, mice were subjected to the social withdrawal test (Figure 8A). The test was performed in dim light, a circular open field, and recorded for 10 min, following a protocol previously described (Koványi et al., 2016). Briefly, two unfamiliar mice receiving the same pharmacological treatment were placed in a circular open field. Mice were placed on opposite sides of the open field. The following behavioral variables were recorded: distance traveled, social interaction, ataxia, and stationary stereotyped behavior. Line crossings and social interactions were recorded for the entire duration of the test using a computer-based event recorder. Social investigations were defined as sniffing and nosing when the nose of the scored mouse touched (or was very close to) the body of the partner and followed toward the partner. PCP-induced stereotyped behavior and ataxia were scored manually according to the protocol described by Nabeshima et al. (1987) and Sams-Dodd (1995), respectively.

#### Subchronic Treatment

P2rx7^+/+^ and P2rx7^−/−^ 2-month-old male mice were housed in individual cages in the behavioral unit and randomly assigned to PCP or vehicle subchronic treatment. On day 3 of the treatment, 90 min after the injection, four randomly selected animals per group were placed into a glass cylinder (10 cm diameter) and recorded for 5 min in dim light. The tests were repeated the day after the fourth injection. After 3 days of treatment washout (72 h from the last injection), the animals were tested in a Y-maze (custom-built: arm length 30 cm; width 7 cm; walls height 20 cm; angle 120° equal) for 10 min at dim light and recorded.

One P2rx7^−/−^ animal acutely treated with PCP was excluded from the analysis as it escaped the cage during the experiment. Subchronically treated animals that did not reach 20 alternations in the Y-maze were excluded. Alternations were considered when an animal changed from visiting one arm to visiting a second arm, with the central body point over 20% of the arm length. Repeated passages from the center of the maze to the same arm were considered a single-arm exploration. Behavior was quantified and analyzed offline using Noldus EthoVision XT^®^.

### Histology

#### Acute Treatment

To study the acute effect of PCP on *in vivo* neuronal activity during the psychotomimetic phase (40–90 min after the injection), animals were sacrificed after reaching the peak of c-Fos IEG protein expression, 90 min after the neuronal activating event (Chaudhuri et al., 2000; Arime and Akiyama, 2017). Therefore, 180 min after the treatment, each animal received 100 mg/kg pentobarbital i.p. dissolved in saline (Nembutal, Sigma–Aldrich Kft, Budapest, 10 ml/kg injection volume in 0.9% NaCl sterile). After 10–20 min, the animal was transcardially perfused for 3 min with saline (flow rate 5 ml/min), followed by 15 min perfusion with 4% paraformaldehyde (PFA, Merck-Sigma) in 0.1 M phosphate buffer (PB, Na_2_HPO_4_ · 2H_2_O; NaH_2_PO_4_, Merck-Sigma) solution. To obtain comparable c-Fos staining, the time window was strictly respected. Following fixation, the brain was rapidly removed from the skull and post-fixed in 4% PFA in PB at 4°C overnight. The following day, the collected brains were extensively washed in PB. The prefrontal cortices were coronally sliced (40 μm thickness, Vibratome Leica VT 1200 Wetzlar, Germany) and rinsed in PB before staining. Coronal slices of the PFC (Bregma +1.70 − +2.10) were selected for immunohistochemistry (Franklin and Paxinos, 1997).

#### C-Fos DAB

Two slices from three saline- and four PCP-treated animals per genotype (P2rx7^+/+^ and P2rx7^−/−^) were incubated for 10 min in 3% H_2_O_2_ in PB, rinsed three times for 10 min in PB, and three times for 10 min in PB. The pre-made blocking buffer (ImmPRESS UNIVERSAL REAGENT, Vector Laboratories, MP-7500) was supplemented with 0.5% Triton X-100 (Tx, Merck-Sigma), and 7.5% of normal donkey serum (NDS, Jackson Immunoresearch, Europe). After 1 h of blocking, slices were incubated with the primary rabbit anti-c-FOS antibody (1:1,000, Santa Cruz Biotechnology sc-52, Dallas, TX, USA) in PB containing 0.05% sodium azide (Merck-Sigma) for 24 h at room temperature (RT) and 72 h at 4°C. Eventually, the slices were rinsed in PB and incubated with the premade secondary anti-mouse/rabbit HRP-conjugated ImmPRESS UNIVERSAL REAGENT (Vector Laboratories, MP-7500) for 1 h. DAB was developed using the commercially available DAB-REACTION KIT (Vector Laboratories, SK-4105) according to the manufacturer’s instructions.

#### Fluorescence Immunohistochemistry

A similar protocol to the above DAB staining was performed for single fluorescence immunostaining for c-Fos staining (P2rx7^+/+^ and P2rx7^tg/+^, four and five PCP-treated animals respectively) and double fluorescence immunostaining. Slices were rinsed in PB three times for 10 min and blocked for 1 h (10% NDS; 0.5% Tx in PB). Antibody against murine c-Fos (1:1000, 226.004 Synaptic System, Göttingen, Germany) was dissolved in PB-0.05% sodium azide, 0.1% NDS, and 0.2% Tx. Slices were incubated with the c-Fos primary solution for 1 day at RT and 2 days at 4°C. On the 3 day, the primary antibodies against murine Parvalbumin (PV, 1:500, PVG-213, Swant, Marly, Switzerland), against murine P2Y12R (1:400, AS-55043, AnaSpec, San Jos, CA, USA), NeuN (1:400, MAB377, Merck Millipore, Darmstadt, Germany), and anti-tyrosine hydroxylase (TH, 1:400, EP1532Y, Abcam, Cambridge, UK) were added to the wells for an additional 24 h. Eventually, the slices were rinsed with PB (three times 10 min) and incubated for 1–2 h at RT with the corresponding secondary antibodies (1:500, 706-605-148 Alexa Fluor 647 Donkey anti-guinea pig for c-FOS; 711-605-152 Alexa Fluor 647 Donkey anti-rabbit for TH;705-585-003 Alexa Fluor 594 Donkey anti-goat for PV; 711-545-152 Alexa Fluor 488 Donkey anti-rabbit for P2Y12R, Jackson Immunoresearch, Europe). For the demonstrative co-localization of P2X7R and microglia, PFC slices from P2X7-EGFP mice were rinsed in PB three times for 10 min and blocked for 2 h (5% NDS; 0.3% Tx in PB). The primary antibodies against murine P2Y12R (1:400, AS-55043, AnaSpec, San Jose, CA, USA) and chicken anti-GFP (1:500, GFP-1020, Aves Labs) were dissolved in PB, 5% NDS, 0.3% Tx. Slices were incubated with the primary antibody solution overnight at 4°C. On the 2 day, the slices were rinsed with PB three times for 10 min and incubated for 2 h at RT with the corresponding secondary antibodies (1:500, 711-545-152 Alexa Fluor 488 Donkey anti-rabbit and A-11039 Alexa Fluor 488 goat anti-chicken).

After DAB-revelation or secondary antibody incubation, slices were rinsed in PB three times for 20 min before being mounted on microscopy slides and coverslipped (Thermo-Fisher Scientific) with ProLong™ Gold Antifade Mountant (P36934, Thermo-Fisher Scientific) and kept at 4°C.

### Microscopy

Microscopy was carried out at the Nikon Microscopy Center in the Institute of Experimental Medicine at the Hungarian Academy of Science. Pictures were taken with a confocal Nikon C2 microscope (20× and 60× -oil immersion objectives). Fluorescent picture z-stacks: 11/20 steps z-steps 0.5–1 μm (c-Fos, c-Fos/PV; c-Fos/TH; c-Fos/P2Y12R/NeuN); 30–38 steps z-steps 1 μm (c-Fos/P2Y12R). Bright field c-Fos DAB pictures were taken with a color camera (DS-Fi3), 11 steps per z-steps 2.5 μm. Fluorescent z-stack microscopy images were analyzed using the software FIJI ImageJ (Schindelin et al., 2012). c-Fos counting: “Sum of the slices” (DAB) or “Maximal projection” (fluorescence) images were divided into regions of interest, and the individual layers were analyzed (layers width: *I* = 120–140 μm; II/III = 80 μm; *V* = 320–350 μm; VI = minimum 280 μm, Figure 3A right). The c-Fos positive nuclei mean gray values from the original images were measured over the auto-threshold-counted objects with automated macro processing in FIJI ImageJ. The 2D/3D Sholl analysis was performed using the FIJI ImageJ application (Ferreira et al., 2014).

### [^3^H]-Dopamine ([^3^H]-DA) Release Experiment

The [^3^H]-DA release experiments were conducted using the method described in our previous articles (Csölle et al., 2013; Koványi et al., 2016). Briefly, 2–3-month-old male mice (*n* = 32 in P2rx7^+/+^, *n* = 11 in P2rx7^−/−^and *n* = 4 in P2rx7^tg/+^), were anesthetized under light CO_2_ inhalation, decapitated, and the brain was extracted from the skull. The PFC dissected in ice-cold Krebs’ solution saturated with 95% O_2_ and 5% CO_2_. Coronal 400 μm-thick PFC slices were chopped (McIlwain tissue chopper) and incubated for 45 min at 37°C in 1 ml Krebs solution with 5 μCi/ml [^3^H]-DA (specific activity 60 Ci/mmol; ARC, St. Louis, MO, USA) bubbled with 95% O_2_ and 5% CO_2_. Once loaded with [^3^H]-DA, the slices were continuously superfused with 95% O_2_, and 5% CO_2_-saturated modified Krebs solution (flow rate: 0.7 ml/min). After a 90 min washout period, perfusate samples were collected over 3 min periods and assayed for tritium content. The temperature was maintained at 37°C. At the 20th min, after starting to collect samples, the slices were subjected to 3 min of 20 μM veratridine (Sigma Chemical Co., St. Louis, MO, USA) perfusion. The radioactivity of the samples was measured using a Packard 1900 Tricarb liquid scintillation spectrometer, using an Ultima Gold Scintillation cocktail. The release of tritium was expressed as a percentage of the amount of radioactivity in the tissue at the sample collection time (fractional release). The tritium uptake in the tissue was determined as the sum of release + the tissue content after the experiment and expressed in Bq/g. For the evaluation of the basal tritium outflow, the fractional release measured in two consecutive 3 min samples under drug-free conditions was considered. The veratridine-induced [^3^H]-DA efflux was calculated as the net release in response to the respective stimulus by subtracting the release before the stimulation from the values measured after stimulation.

### Electrophysiology

Eight P2rx7^+/+^ and six P2rx7^−/−^ male mice 60–75 days old were anesthetized with isoflurane (IsoVetR 469860, Braun) and decapitated. The brain was extracted, put on ice and immediately immersed in ice-cold *N*-methyl D-glucamine based cutting solution (NMDG 92 mM, NaHCO_3_ 30 mM, NaH_2_PO_4_ 1.25 mM, HEPES 20 mM, glucose 25 mM, Na-ascorbate 5 mM, Na-pyruvate 3 mM, Thiourea 2 mM, KCl 2.5 mM, MgSO_4_ 10 mM, CaCl_2_ 0.5 mM; all from Merck-Sigma; pH 7.3) for 2 min, before being embedded in 2% agarose gel (Merck-Sigma) and sliced at 300 μm thickness on a Compresstome (VF-300-0F Precisionary). Coronal PFC slices (Bregma +1.70 − +2.50) recovered for 30 min in an NMDG-based cutting solution bubbled with 95% O_2_ and 5% CO_2_ at 37°C. Slices were transferred for an additional hour in aCSF (300/306 mOsm, pH 7.3, NaCl 119 mM, KCl, 2.5 mM, MgCl_2_ 1.6 mM NaHCO_3_ 26 mM, NaH_2_PO_4_ 1 mM, HEPES 5 mM, D-glucose 10 mM, CaCl_2_ 2.5 mM; Merck-Sigma) at RT. Patch-clamp recordings of neurons in the prelimbic mPFC cortical layer V were made using infrared differential interference contrast video microscopy (CleverExlore, MCI Neuroscience). For whole-cell patch-clamp recordings, a pipette solution containing K-gluconate 125 mM, KCl 5 mM, HEPES 10 mM, EGTA 1 mM, 4 mM Na_2_GTP, 0.3 mM, NaP-creatine 10 mM, ascorbic acid 3 mM (280–295 mOsm, pH 7.3; all from Merck-Sigma) was used, yielding a tip resistance of 4–5 MΩ. Recordings were obtained 200 s after the entire patch-clamp configuration to allow steady-state conditions between the pipette solution and the cytosol. The resting membrane potential (Vm) was recorded in current-clamp mode at 0 pA for 10 s. Current/Voltage (I/V) relationship and action potential firing were obtained with 0.4 s square pulse current step injections from −100 pA to +280 pA at 20 pA increments (I step, 1 per second). At 100 ms before Istep, a 1 ms current pulse at −75 pA was injected to normalize the Vm after depolarization. The membrane potential at 0 pA before and after Istep, were taken from the average voltage of the 30 ms before Istep (Vm pre) and of the last 0.1 s after 0.3 s from I step (Vm post). Ramp injection of 0–500 pA current over 1 s was used to determine the current required to elicit an action potential, that is, rheobase. All currents and voltages were registered and controlled using a HEKA EPC10 amplifier. Data were analyzed using the Fitmaster software (HEKA electronic).

### Cytokine Quantification

Cytokine quantification from the PFC of subchronically treated animals was performed as described in our previous article (Horváth et al., 2019). Briefly, on day 14, after the first PCP or vehicle injection, brain samples were collected after light CO_2_ anesthesia. Tissue was homogenized and centrifuged, as described previously (Dénes et al., 2010), and supernatants were collected to measure the levels of the following inflammatory mediators: IL-1α, IL-1β, IL-10, TNF-α, and CXCL1 (KC) using BD Cytometric Bead Array Flex Sets (BB Biosciences). Measurements were performed on a BD FACSVerse flow cytometer, and data were analyzed using FCAP Array version 5 (Soft Flow). Cytokine concentrations in the brain tissue were normalized to total protein levels measured by photometry using a BCA Protein Assay Kit (Thermo Fisher Scientific, Pierce). Absorbance was measured at 560 nm using a Victor 3V 1420 Multilabel Counter (PerkinElmer). Plasma cytokine levels are expressed as picograms per milligram.

### Statistics

Statistical analyses were performed using STATISTICA version 64 (StatSoft Inc., Tulsa, OK, USA) and Graph Prism version 5 (GraphPad Software, San Diego, CA, USA). The RT-qPCR data were analyzed using the − delta-delta Ct (2^−ΔΔCt^) calculation method, for comparison was used unpaired nonparametric Mann–Whitney *U* test. Differences between two groups were analyzed using the Student’s *t*-test, between multiple groups one-way analysis of variance (ANOVA) followed by a *post hoc* Kruskal–Wallis or Dunn’s comparison test or two-way ANOVA followed by Bonferroni’s *post hoc* test or Mann–Whitney *U*-test, as appropriate for multiple comparisons was used. The specific tests used are reported in the captions of the figures. All data are expressed as a mean ± standard error of the mean (SEM; *p < 0.05, **p < 0.01, ***p < 0.001, ****p < 0.0001). A *p*-value of lower than 0.05 was considered to be statistically significant.

## Results

### Acute PCP Treatment-Induced Hyperactivity and Layer-Specific Neuronal Activation in the mPFC Was Alleviated in P2rx7^−/−^ Mice

To test the validity of the P2X7R knockout model, real-time qPCR (RT-qPCR) reaction detecting P2rx7 gene transcripts was performed (Figure 1A). The RNA has been extracted from the whole frontal cortex (Figure 1B). The RT-qPCR measurement revealed that the disrupted sequence in P2rx7^−/−^ mice was markedly reduced compared to the P2rx7^+/+^ mice, while no difference was detected regarding the extra- and intracellular sequences between the knockout and WT mice (Figure 1C). The visualization of RT-qPCR products by electrophoretic separation confirmed that the band (90 bp), corresponding to the disrupted sequence in P2rx7^−/−^ mice, appeared only in the P2rx7^+/+^ (data not shown), while the bands corresponding to the extra-, and intracellular regions of P2X7 receptor (at 389 bp and 58 bp, respectively) were detected in both the WT and in the KO mice (data not shown). Implications of the presence of P2rx7 transcripts will be further elaborated in the “Discussion” section. For sake of clarity, in the current text, this model is referred to as the genetically deficient mouse strain for the P2X7 receptor (P2rx7^−/−^).

To evaluate the involvement of P2X7R in the PCP-induced hyperactivity of the mPFC circuit, locomotor activity in an open-field arena was evaluated, and neuronal activation was monitored using c-Fos immunohistochemistry. The proto-oncogene c-Fos is a transcription factor responsible for the formation of Activator-Protein-1 (AP-1) together with c-Jun. It is widely accepted that c-Fos activation and neuronal activity directly correlate (Day et al., 2008). Every animal followed a strict schedule from the beginning of the behavioral protocol until brain extraction (Figure 2A).

P2rx7^+/+^ and P2rx7^−/−^ mice were treated with 10 mg/kg PCP i.p. and immediately placed in a new cage. PCP increased locomotion and elicited stereotypic behavior. Hyperlocomotion and rotational stereotypic behavior peaked between 40 and 80 min after injection (Figures 2B,C).

The effects of PCP in P2rx7^+/+^ animals were more pronounced in terms of stereotypy and longer-lasting hyperlocomotion than P2rx7^−/−^ mice (Figure 2C). Along the mPFC of treated animals, PCP induced a clear band of c-Fos immuno-positive neuronal somata, primarily at the level of layer V (Figure 3A). The ventrodorsal gradient of active neuron concentration revealed a robust PCP-driven engagement of the infralimbic (IL) and prelimbic (PL) areas (Figure 3B). P2rx7^−/−^ mice displayed reduced PCP-induced hyperlocomotion and stereotyped behavior (Figure 2C). This was accompanied by a lower number of c-Fos-positive nuclei in the ventral region of Layer II/III, but not Layer V (Figure 3B). Since the number of c-Fos-positive cells and the level of c-Fos protein expression was established as a neuronal activity marker (Chung, 2015), a deeper inspection of the IL and PL areas in PCP-treated mice was performed. Two different anti-c-Fos antibodies with different protocols were employed to enhance reliability (Figures 3A,C). Plotting the number of c-Fos-positive neurons against the intensity of the signal revealed different distribution between genotypes (Figure 3D). Pragmatic discrimination between activated (c-Fos^+^) and strongly activated (c-Fos^++^) neurons, setting the dimension and intensity threshold to 200 μm^2^ and 400 mean gray value (ImageJ standardized units), respectively, between c-Fos^+^ and c-Fos^++^, revealed a significant reduction in the concentration of c-Fos^++^ neurons in the P2rx7^−/−^ IL and PL areas, specific to layer V (Figure 3E). These results suggest that the genetic deletion of P2X7R in part buffers the psychotomimetic effects of PCP and the consequent layer-specific neuronal activation in the mPFC.

### P2X7R Deficiency Does Not Affect PCP-Related Changes in PV Interneurons in the PFC and Dopamine Release From the Striatum

To determine the underlying mechanism involved in the differential effect of acute PCP in P2rx7^+/+^ and P2rx7^−/−^ animals, double immunohistochemistry for c-Fos, and PV were performed, and microscopic images were manually analyzed by an investigator blinded to the treatments and genotype (Figure 4A). No difference was detected between the genotypes in either the total number of PV positive interneurons (Figure 4B) or in the percentage of those activated by systemic PCP (Figure 4C) in the layers II/III and V of the mPFC. Next, we explored the influence of P2X7R deficiency on the density of dopaminergic fibers. We did not observe major genotype-related anatomical differences by analyzing the double immunostaining for the c-Fos increase and tyrosine hydroxylase (TH, dopamine- and noradrenaline-synthesizing axon terminals marker) of the mPFC (Figure 5A). In this preliminary study, longitudinal quantification of signal intensity confirmed an increase in c-Fos expression (Figure 5B, upper panel) in wild-type PCP-treated animals within layer V (200/220 μm lateral to the midline). At the same time, there was no change in TH positive fiber density (Figure 5B, lower panel, and Figure 5C). Next, we have studied the release of dopamine with tritiated dopamine from the local dopaminergic afferents in the PFC to clarify the involvement of P2rx7^−/−^ or P2rx7^tg/+^. Analysis of 20 μM veratridine-induced (Fekete et al., 2009), Na^+^ channel-mediated tritiated dopamine release (perfusion for 3 min leads to a transient Na^+^ channel activation, Figure 5D) did not differ between genotypes in both sets of experiments. Confirming our previously published results (Koványi et al., 2016), in the PFC of P2rx7^−/−^ animals, the basal dopamine release was lower (Figure 5E, left panel). On the other hand, dopamine uptake of P2rx7^tg/+^ PFC was lower in comparison to the corresponding wild type controls (Figure 5F, right panel).

### P2X7R Gene Deficiency may Play a Role in Microglial Contacts With Hyperactive Neuronal Soma

Next, we addressed the role of the P2X7R expressed by microglial cells in the context of PCP-driven neuronal activation. To label microglia, P2Y12 receptor (P2Y12R) immunohistochemistry was used, which is widely used as a marker of resting, but not activated microglia (Haynes et al., 2006; Cserép et al., 2020), and is a part of the exclusive microglia signature (Butovsky et al., 2014; Calovi et al., 2019).

Coronal IL and PL cortical layer V immunohistochemical staining against P2Y12R (Figures 6A,C) or P2Y12R and c-Fos (Figures 6G,I) from P2rx7^−/−^ and P2rx7^+/+^ animals treated with saline or PCP were performed. Moreover, additional triple immunohistochemistry, with the inclusion of the NeuN (neuronal nuclear marker) of slices from PCP-treated animals (Figure 6E), was performed. Manual and automated analyses showed no difference in microglial density and general ramification by treatment or genotype, while a slightly smaller P2Y12R immunoreactivity was noted in P2rx7^−/−^ concerning P2rx7^+/+^ after acute PCP (Figure 6B). Maximal projections (10 μm thick stacks) of IL and PL areas stained for P2Y12R allowed us to perform a soma-centered 2D-Sholl analysis over non-isolated microglial cells (Figure 6C, left). Thus, this analysis does not present the characteristic “crossing branches count” curve from single-cell ramifications. Microglia ramification in the cortex shapes an intricate network of processes, in which single cells marked by a unique morphology can extend up to tens of microns, rendering complex reconstruction and isolation of cellular microglia in a 3D space. This novel analysis returned in a mixed Sholl profile where, instead of a bell-shaped curve, the number of crossing branches increased linearly with the extension of the Sholl radius (Figure 6D). It is reasonable to consider that the area included by small Sholl circles (0–20 μm radius, red bar Figures 6C,D) usually represents the ramification of a single cell since microglial nuclei are evenly distributed in the cortex. Bigger Sholl circles (20–40 μm radius, green bar Figures 6C,D) cover the territory patrolled by several cells, therefore representing an inter-cellular territory (Figure 6C, right panel). Adopting as the *y* Cartesian axis graph the graph identity function (*f*_(x)_ = *x*, red-dashed line Figure 6D) allows better visualization of differences in the microglial ramification profiles between the groups (Figure 6D″). This illustrates that the difference between groups was not related to single-cell ramifications (Figure 6C; red bar), while P2rx7^−/−^ microglial processes were significantly denser in the intercellular territory (Figure 6C, left panel; green bar, **C**, right panel; the green-colored area, **D"**; green bar). Genotype-related differences faded with acute PCP treatment (Figure 6D″).

To better understand the relationship between neurons and microglia in the context of PCP-driven hyperactivation, mPFC slices were triple-immunostained for P2Y12R, NeuN, and c-Fos (Figure 6E). Neurons whose nuclei were NeuN^+^ and c-Fos^−^ were considered to be not recruited by the PCP-driven effect and are herein referred to as inactive neurons. We examined whether the guidance of microglial branches toward active nuclei would be detectable by applying the aforementioned 2D Sholl analysis, this time centered in active or inactive neurons of the mPFC layer V (Figure 6E). We confirmed that c-Fos^+^ neuronal nuclei, both in the analyzed P2rx7^−/−^ and P2rx7^+/+^ PCP treated animals, are surrounded by a higher number of microglial branches compared to inactive neurons in the same field of view (Figure 6F). While revealing a significant treatment effect, the 2D Sholl analysis did not detect differences between genotypes concerning microglial branches surrounding the nearby parenchyma of both inactive and activated neurons (Figure 6F″).

As for microglia organization concerning c-Fos positive IL and PL neurons in PCP-treated animals (Figure 6G), we noticed that in P2rx7^−/−^ mice, the strongly activated neurons (c-Fos^++^) tended to be more often in contact with microglial somata. However, this was not statistically significant (Student’s *t*-test *p* = 0.0967, Figure 6H). To obtain a precise quantification of the microglia branches in the proximity of hyperactive neurons, larger z-stacks over 30 μm thick were taken. A 3D Sholl analysis of microglial staining, centered on c-Fos^+^ nuclei, was performed (Figure 6I). The 3D Sholl analysis, performed over a radius of 15 μm, revealed a greater number of microglial branches recruited by c-Fos^+^ neurons in the P2rx7^−/−^ prefrontal cortical layer V (Figure 6J).

### Intrinsic Properties of P2rx7^−/−^ mPFC Layer V Pyramidal Neurons

To investigate whether the difference in PCP-driven c-Fos immunoreactivity could be related to congenital/developmental intrinsic hypoexcitability of neurons in P2rx7^−/−^ animals, patch-clamp recordings of mPFC layer V neurons were performed (Figure 7A). To reduce developmental variability, neurons from mice older than 59 days were recorded.

Thirteen neurons from eight P2rx7^+/+^ animals and 21 neurons from six P2rx7^−/−^ animals were patch-clamped and analyzed. No difference in series resistance (35.7 ± 3.2 MΩ P2rx7^+/+^; 33.4 ± 2.4 MΩ P2rx7^−/−^) and membrane resistance (375.6 ± 38.7 MΩ; 433.5 ± 56 MΩ) were found between the genotypes. Similarly, no difference in resting membrane potential recorded at 0 pA current-clamp was observed (Figure 7B). As measures of excitability, rheobase and IV relationships were recorded in current-clamp mode using the ramp and step protocols, respectively. We found no difference in these values between P2rx7^−/−^ and P2rx7^+/+^ control animals (Figures 7C,D). Following action potential-induced depolarization, P2rx7^−/−^ PFC neurons displayed faster repolarization of the membrane potential than wild-type (red segment Figures 7A,E). Moreover, during depolarizing current injections, P2rx7^+/+^ neurons fired more action potentials than P2rx7^−/−^ neurons at the same current pulse, indicating a difference in spike accommodation (Figure 7F).

Our data confirm that in mPFC layer V of P2rx7^−/−^ and P2rx7^+/+^, pyramidal neurons of young adult C57Bl/6J animals present similar input-related response characteristics, and no difference in the current amount necessary to reach the action potential threshold (rheobase). We found that P2rx7^+/+^ neurons respond more robustly to membrane depolarization than P2rx7^−/−^. Wild-type neurons present a shorter refractory period, therefore firing a higher number of action potentials, and has longer-lasting depolarization of the membrane after action potential firing. This indicates that P2rx7^−/−^ could be less responsive to strong neuronal activation.

### P2X7R-EGFP Overexpressing Reporter Mice Displayed Higher Sensitivity to Acute Low-Dose PCP Treatment

Our work conducted on P2rx7^−/−^ and P2rx7^+/+^ suggests that P2X7R deficiency ameliorates acutely induced PCP psychotomimetic hyperactivity in the mPFC, likely involving lower basal dopamine release, increased microglia-hyperactive neuron interaction, and faster neuronal repolarization after action potential firing. These findings collectively result in lower activation of a subgroup of layer V mPFC neurons.

To corroborate these findings in P2X7R knockout animals, it is crucial to test an alternative animal model to evaluate possible predominant collateral artifacts, such as developmental abnormalities.

Therefore, we carried out a behavioral study on a recently developed mouse model, that is, the heterozygous C57Bl/6J mouse line overexpressing the P2X7-EGFP protein (Kaczmarek-Hajek et al., 2018), referred to as P2rx7^tg/+^. To assess acute PCP-susceptibility, P2rx7^tg/+^ and P2rx7^+/+^ mice were subjected to a low-dose of PCP (2 mg/kg i.p.) followed by a modified protocol of the open field and social withdrawal tests, previously validated for PCP treatment evaluations. Their behavior was recorded over a 10 min trial period (45 min after the PCP or vehicle, Figure 8A) and analyzed, as previously described (Koványi et al., 2016).

Basal levels of locomotion, social behavior, stereotypical behavior, and ataxia were not different between the genotypes, while PCP treatment affected several behavioral parameters (Figure 8B). In P2rx7^+/+^, acute PCP treatment strongly diminished social interaction and promoted stereotypical behavior (Figure 8B, left-center panel, and right-center panel). However, this lower dose of PCP did not significantly alter the locomotion of P2rx7^+/+^ animals and failed to trigger ataxia (Figure 8B, left and right panels). In contrast, the acute PCP effect involved all tested behavioral parameters in P2rx7^tg/+^ mice, which exhibited hyperlocomotion and features related to ataxia already appearing in some mice (Figure 8B, left and right panels).

To visualize the P2X7R protein expression in the mPFC, we have performed immunostaining against P2X7R-EGFP and the purinergic receptor P2Y12R, using the P2rx7^tg/+^ reporter mice. Figure 8C shows the overall P2X7R expression pattern (violet), by immunostaining against the EGFP part of the P2X7R and the P2Y12R (green). The presence of the receptor is conspicuous in the microglia of this mice model, yet interestingly we observed EGFP positive/P2Y12R negative fibers as well, implying that the receptor is also expressed by other neural cell types (Figure 8C).

### Acute PCP Treatment-Induced Hyperactivity and Layer-Specific Neuronal Activation in the mPFC Was Exacerbated in P2rx7^tg/+^ Mice

To corroborate the involvement of P2X7R in the PCP-induced hyperactivity of the mPFC circuit, the acute PCP experiment was repeated similarly (normal light), this time testing the P2rx7^tg/+^ mouse line (Figure 9A).

PCP increased locomotion and elicited stereotypic behavior. Hyperlocomotion and rotational stereotypic behavior peaked between 30 and 70 min after injection (Figures 9B,C).

The effect of PCP in P2rx7^tg/+^ animals were strongly pronounced in terms of stereotypy in respect to the P2rx7^+/+^ mice (Figure 9C). Fluorescent c-Fos immunostaining of treated animals in both analyzed bregmas showed the typical band of c-Fos immuno-positive neuronal somata corresponding to the layer V of the IL and prelimbic PL areas (Figure 9D).

A similar analysis to the one performed in Figure 3 was performed on 5 μm thick stacks maximal projections images, focusing on the layer V of the IL and PL areas of PCP-treated mice. The exacerbated stereotypy of P2rx7^tg/+^ mice was accompanied by a higher number of strongly activated (c-Fos^++^) positive nuclei, specific to the Bregma 1.70 but not to the Bregma 2.10, in the IL and PL region of the Layer V (Figure 9E).

In this case, the dimension and intensity threshold to count c-Fos^++^ neurons were set to 100 μm^2^ and 1,100 mean gray value (ImageJ standardized units), respectively, selected according to the visual comparison with the previously selected nuclei. These results suggest that the overexpression of P2X7R has opposite effects concerning the receptor-deficient model, and exacerbated the psychotomimetic effects of PCP and the consequent layer-specific neuronal activation in the mPFC.

### Subchronic PCP Failed to Induce Working Memory Deficits in P2X7R Deficient Animals

We decided to evaluate the possible impact of P2X7R deficiency on the cognitive symptoms induced by repeated PCP administration (daily 10 mg/kg i.p. per 7 days, Zain et al., 2018) with a simple battery of behavioral tests (Figure 10A).

Before the first treatment, P2rx7^−/−^ animals had a higher body mass compared to the P2rx7^+/+^ animals (Giacovazzo et al., 2018), which normalized after the 1 day (Figure 10E).

To confirm whether repeated PCP injections did not induce long-lasting alterations in basal locomotor activity, four animals per group were tested in a confined environment, a glass cylinder, 90 min, and 20 h after receiving the third PCP or vehicle treatment. After drug clearance, no difference in locomotor behavior was observed (Figure 10B).

At the end of the subchronic PCP or vehicle treatment, animals experienced 72 h of withdrawal before being tested in a Y-maze. We were particularly interested in assessing the status of the working memory. The working memory is strictly dependent on the mPFC microcircuit integrity, and at the same time, is a major cognitive symptom of SCZ that is still largely untreated by available treatments (Elsworth et al., 2014).

Both PCP and vehicle-treated P2rx7^−/−^ animals covered a significantly shorter distance during the trial, yet the number of total alternations was not affected by either genotype or treatment (Figure 10C, left and center panels).

P2rx7^+/+^ PCP-treated mice tested for spontaneous alternations displayed fewer alternations concerning the P2rx7^+/+^ and P2rx7^−/−^ PCP-treated animals. The scores of P2rx7^−/−^ animals were unaffected by subchronic PCP, indicating that subchronic PCP failed to induce working memory deficits in P2X7R deficient animals (Figure 10C, right panel).

After two additional days of washout, an open field test was performed for 10 min on each animal, which did not show any influence by either the genotype or treatment in locomotor activity (Figure 10D).

Finally, we quantified cytokine levels in the brain, considering that neuroinflammatory events are accompanied by changes in cytokine concentrations, and are reportedly depend on P2X7R-mediated mechanisms (Di Virgilio et al., 2017; He et al., 2017).

A significant constitutive increase in the fractalkine ligand (CX3CL1) level was found in the P2rx7^−/−^ PFC, after both PCP and vehicle subchronic treatments, whereas the levels of other measured cytokines were not affected by either the treatment or genotype (Figure 10F). The effect of subchronic PCP on IL-1β was not significantly different between the two genotypes or when expressed as a percentage of the control (Figure 10F″).

Our subchronic PCP experiments, which suggest a beneficial role of P2X7R deficiency in the context of the PCP-induced loss of working memory in rodents, do not point to a major change in the consequent neuroinflammatory phenomenon in the prefrontal region.

## Discussion

In this study, we found that the effect of PCP in mice is modulated by P2X7R in terms of positive and cognitive symptoms. Genetically deficient animals for the receptor displayed a lower susceptibility to the psychotomimetic effects of the dissociative anesthetic, while the overexpression of the purinergic receptor produced a typically positive symptom response within a sub-optimal PCP dose for psychosis-like activity (Paasonen et al., 2017) and exacerbated stereotypy with an acute psychotomimetic 10 mg/kg PCP dose. Most importantly, after a subchronic PCP treatment, P2rx7^−/−^ mice did not display a reduction in working memory, typical of the model’s cognitive symptomatology. Before discussing the results obtained with P2rx7^−/−^ mice, it is appropriate to review the current genetic model used (Solle et al., 2001), since it is a source of some controversy. The gene of the P2rx7 receptor protein is located in the mouse chromosome Chr.5, 62.50 cM. Persistent stimulation of P2X7R triggers the ATP-dependent opening of an aqueous plasma membrane pore. Most of the pathophysiological functions of P2X7R are thought to be dependent on this macropore formation, suggested to be an intrinsic property of the channel itself (Di Virgilio et al., 2018). While nine splicing variants are reported in humans, in the mouse four alternative variants that generate four P2X7R subunits have been identified (Sluyter, 2017; Adinolfi et al., 2010). The full-length variant, named P2X7A in human and mouse, is generally found co-expressed with the mouse naturally occurring P2X7k variant, which seems to escape inactivation, representing a gain of function isoform (Kaczmarek-Hájek et al., 2012). Regarding receptor knockout animal models, at least two strains of mice are currently commercially available: a P2X7R knock out strain with reporter function produced by GlaxoSmithKline, in which exon 1 has been replaced with a lacZ gene and neomycin cassette (Neo); and a strain from Pfizer (commercially available from The Jackson Laboratory, the strain used in the current study), in which a portion of the exon 13, encoding Cys506 to Pro532, has been deleted and replaced with a Neo, truncating the long C–terminal cytoplasmic tail (Sikora et al., 1999; Solle et al., 2001). Nonetheless, antibody detection and Ca^2+^ responses indicative of P2X7R functional expression in cerebellar neurons and midbrain synaptosomes from the Pfizer knockout mice line have been reported, yet the pharmacological assays suggest a P2rx7 loss of function in the knockout line (Sánchez-Nogueiro et al., 2005; Marín-García et al., 2008). In addition, whether two C-terminally truncated splice variants of the P2X7R escape the Pfizer Knock out inactivation strategy cannot be excluded; however, currents mediated *via* these C-terminally truncated P2X7 splice variants are of much lower amplitude compared to the P2X7A mediated receptor currents (Adinolfi et al., 2010; Masin et al., 2012). As a result of the presence of the truncated and probably partly functional variant of P2X7 (Sánchez-Nogueiro et al., 2005), it might contribute to the relatively modest amelioration of symptoms in the P2X7-KO mice. Additionally, the Pfizer produced P2X7R knockout mouse line is bred over a C57bl/6, which constitutively co-express the P2X7A and P2X7k splicing variants of the receptor in all the systems. Our RT-qPCR results confirm the presence of P2rx7 RNA in the prefrontal cortex of the Pfizer strain, yet the disrupted portion is found only in wild type tissue suggesting that a COOH truncated isoform with a lowered functionality is expressed by the tested animals.

Our current results confirm and extend our previous observations (Koványi et al., 2016) and others (Lord et al., 2014; Gubert et al., 2016) obtained with genetic deletion and pharmacological inhibition of P2X7R. A significant novel observation of the present study is the alleviation of neuronal activation in a restricted area of the mPFC (layer V of prelimbic and infralimbic areas) in response to PCP treatment in mice genetically deficient for P2rx7, while the opposite effect is obtained with the receptor overexpression. This finding identifies a potential primary site of action, whereby P2rx7 might affect PCP-induced behavior.

The rodent mPFC primarily involved in the PCP psychotomimetic effect is of particular interest since this area is affected by SCZ pathology (dorsolateral frontal cortex for humans) and SCZ models of both positive, negative, and cognitive symptoms are a crucial node for cognitive abilities (Celada et al., 2013). We confirmed that PCP treatment produced a lower number of heavily stained c-Fos neuronal nuclei in the P2rx7^−/−^ genotype, which is an indirect measurement of *in vivo* neuronal activity (Chung, 2015). In the same area, heterozygous P2X7R-EGFP mice displayed an increased concentration of strongly stained c-Fos neurons compared to wild type controls, therefore showing an opposite readout concerning the knockout animals. Our data imply that the difference in P2X7R expression potentially modulates the PCP effect concerning the prefrontal activity in mice. However, we cannot exclude that the P2rx7 genetic manipulations could lead to artifacts, such as altered protein degradation dynamics.

To explore possible protective mechanisms happening in the P2rx7^−/−^, we examined different aspects of the fifth layer of the infralimbic and prelimbic cortices. Our observation from the double and triple immunostaining, [^3^H]-DA release experiments, and electrophysiology were consistent with a possible pleiotropic role of P2rx7 gene deletion as a negative modulator of the PCP psychotomimetic effect.

Differences in PV interneuron activation were excluded from playing a role linked to the lower c-Fos immunoreactivity detected in PCP-treated P2rx7^−/−^ mice. It is worth mentioning that a previous c-Fos study reported an increase in PV interneuron activity in areas involved in acute PCP-induced hyperactivity, but different from the mPFC (Celada et al., 2013; Hervig et al., 2016). The detrimental effect of subchronic PCP models on prefrontal GABAergic interneurons is well documented (Piyabhan et al., 2019).

To rule out developmental abnormalities in our model strain we investigated and found no gross anatomical deviation of the dopaminergic and noradrenergic fibers. Furthermore, P2rx7^−/−^ and P2rx7^tg/+^ displayed no difference in the chemically stimulated local release of tritiated dopamine. We confirmed a small but significant effect on basal dopamine release from the local dopaminergic fibers in the P2rx7^−/−^ PFC (Koványi et al., 2016). Interestingly, in the P2rx7^tg/+^ PFC slices, we could detect a lower tritiated dopamine uptake. These direct measurements may imply a functional role of P2rx7 in regulating basal dopamine local concentration *via* modulating receptor expression. Additionally, dysfunctional P2rx7 expression hints to a deregulated prefrontal dopaminergic tone, which leads to a complex scenario: it is predicted that basal dopamine release and the related compensatory mechanisms in the PFC have an inverted “U” relationship with PFC performance and was shown to be relevant to SCZ symptomatology (Rolls et al., 2008).

We also observed marked change in the microglia morphology at the level of PL and IL layer V, raising the possibility that P2X7 regulates the microglia-neuron interaction, and thereby affects the behavior of the animals.

The number of microglial cells is not altered upon PCP treatment, which is not surprising, considering that migration of microglia cell bodies works on the scale of hours to days (Eyo et al., 2018). Microglia pro-inflammatory activation is also a phenomenon hardly considered to take place in PCP models, except for the Olney’s lesion in the retrosplenial cortex (Olney et al., 1989; Zhu et al., 2014).

We detected a small decrease in P2Y12R immunoreactivity in P2rx7^−/−^ PCP-treated animals compared to the PCP treated wild-type, which could be interpreted as a sign of an initial shift toward a pro-inflammatory profile (Haynes et al., 2006). Nevertheless, no difference was found between the vehicle and PCP-treated groups, indicating that acute neuroinflammatory reactions, if any, are not detected by tissue immunostaining. Indeed, the most convincing information from P2Y12R immunostaining lies in the precise morphological outline of the microglial membrane (Cserép et al., 2020). With unbiased image analysis, using the software FIJI ImageJ, it is possible to obtain an accurate 1-pixel skeleton of the microglia reticule, which renders a Sholl analysis of the skeleton largely independent of immunoreactivity signal oscillations (Schindelin et al., 2012; Ferreira et al., 2014). From the 40 μm thick slices, image stacks of 10–12 μm thickness, starting 5 μm down/up from the slice surfaces, resulted in the best images with the adopted antibodies and protocols. Therefore, the material to analyze was undersized in the z dimension to obtain any single microglial cell ramification fully reconstructed in three dimensions. Indeed, the resulting 3D isolated cells displayed cut branches, and therefore the whole-cell dimension depended on the orientation of its branches with respect to the coronal cut (data not shown). Considering the limitations of the isolated cell analysis, we applied the Sholl analysis on the whole microglia skeleton in the tissue slice. The general microglia ramification (IL and PL cortices, layer V) displayed similar stained areas, suggesting that PCP (10 mg/kg) and P2rx7 gene deletion did not give rise to a plain hyper- or hypo-ramified microglia phenotype.

It was recently reported by Liu et al. (2019), that noradrenaline is the master regulator of microglia general ramification. Two-photon *in vivo* imaging of rodents transitioning from the awake state to the anesthetized state, *via* lowered noradrenergic tone, revealed the expansion of microglial surveillance territory in minutes (Liu et al., 2019). In the current study, we could not detect any PCP-induced anesthesia-like effect on microglial ramification. This observation is in line with the observed acute long-lasting increase, but not decrease, of noradrenergic tone at the level of the rodent mPFC within the PCP psychotomimetic dose range (Kehr et al., 2018). We could not track possible differential effects induced by anesthesia (Nembutal, 100 mg/kg), which probably affected the prefrontal noradrenergic tone in the 20 min following the injection (Pan and Lai, 1995). Since all animals underwent the same procedure, we acknowledge the systematic error.

Microglia cell-centered analysis revealed that microglia of saline-treated P2rx7^−/−^ mice displayed a higher number of branches after the first 20 μm of Sholl radius compared to the saline-treated P2rx7^+/+^ ones. Additionally, PCP treatment dissolves the genotype-related difference in ramification.

It has been reported that P2X7R may retain specific migration and phagocytic properties, as it is essential for CX3CL1 chemoattraction (Fernandes et al., 2016), promote migration toward amyloid senile plaques upon activation while inhibiting microglial phagocytic capacity (Martínez-Frailes et al., 2019), and appears to have a permissive role for NF-κB activation, NLRP3 inflammasome formation and mitochondria toxicity in the microglia (Chiozzi et al., 2019). Regarding the possible role of the P2rx7^−/−^ lower dopaminergic tone, microglia ramification was reported to increase in the presence of a dopamine-3 receptor antagonist (Elgueta et al., 2017), where extracellular dopamine has been reported to exert an anti-inflammatory effect by modulating the inflammasome cascade (Yan et al., 2015). However, we could not find specific information in the literature related to P2X7R expression and microglial ramification.

To match the specific area and location of PCP-driven hyper-activated neurons with microglial branches, we performed double and triple immunohistochemical staining of the mPFC of the treated animals.

Microglial branches are in continuous movement and dynamically contact and explore the surrounding parenchyma concerning external stimuli, as in the case of purine-driven chemotaxis (Calovi et al., 2019). However, recent evidence demonstrates that around neuronal bodies, microglial contacts are a rather specialized communication route that is stable for a few tens of minutes (Cserép et al., 2020). They have been proposed to function as a restraint of neuronal hyper-activation (Sharma et al., 2020). From the PCP-related event that drives mPFC neuron hyperactivity until brain fixation (maximal cumulative c-Fos expression), it took 110 min, which should outdistance the neuronal hyper-activity-related recruitment of microglial branches around neuron somata. The neuron-centered 2D Sholl analysis revealed that microglial branches were more abundant in the proximity of c-Fos positive neurons relative to the negative ones in both genotypes. We observed that the contacts between microglial branches and hyperactive neurons *in vivo* might be stable within a few hours, rather than tens of minutes, in specific pathological contexts. A series of c-Fos and P2Y12R immunostained images with a minimum 30 μm z-stack thickness were taken to perform a 15 μm Sholl radius 3D analysis centered in c-Fos^+^ nuclei located in the central 15 μm along the z-axis. Within the 15 μm radius sphere, in PCP-treated P2rx7^−/−^ animals layer V mPFC hyper-activated neuronal somata were contacted by one to two more microglial branches (approximately 9–16%) than corresponding neurons in wild-type animals. This difference may reflect an increased tendency of P2rx7^−/−^ c-Fos^+^ neuron somata to be in direct contact with microglia somata.

Microglia-neuron contacts are an intriguing subject that recently started to be molecularly characterized. However, it is still too early to conclude any relationship between P2rx7 expression and microglial behavior. Moreover, it is important to verify whether intrinsic neuronal properties could explain the differences observed between the genotypes since neurons can directly attract microglial branches. Patch-clamp recordings of mPFC layer V neurons identified no functional differences between genotypes concerning resting membrane potential or rheobase. However, in the current clamp pulse step protocol, we found a difference in spike accommodation and re-polarization, with an increased firing rate and slower re-polarization to resting membrane potential in wild-type animals. This identifies the subtle excitatory effect of P2rx7 on these neurons.

The role of P2X7R during development seems to be commonly related to necrosis and apoptosis (Kanellopoulos and Delarasse, 2019), yet some *in vitro* studies have pointed out different possible regulatory effects in axonal growth and branching (Díaz-Hernandez et al., 2008). The functionality of these receptors in early and neuronal development remains unknown, as current studies are mainly focused on adults and aging. To our knowledge, this is the first report of patch-clamp analysis on young adult P2rx7^−/−^ prefrontal IL and PL layer V neurons. The electrophysiological profile of P2rx7^−/−^ neurons is in line with our c-Fos results, in that P2rx7^+/+^ mPFC layer V neurons were prone to fire a higher amount of action potential for the same amount of current compared to P2rx7^−/−^ neurons. We cannot exclude that this difference is general to more areas of the mouse brain. If the diminished P2rx7^−/−^ neuronal excitability corresponds to a lower PCP-driven mPFC neuronal hyperactivity (c-Fos signal), it is surprising to observe that a higher number of microglial branches were attracted to the P2rx7^−/−^ strongly activated neuronal bodies. Indeed, microglia/neuron interaction is supposed to be driven by ATP/ADP neuronal release, which has been observed as dependent on neuronal activity (Cserép et al., 2020; Sharma et al., 2020). In contrast, the higher number of attracted microglial branches could also be a compensatory phenomenon. A better characterization of *in vivo* or *ex vivo* microglial dynamics could shed further light on the significance of this observation.

We observed that P2rx7^tg/+^ displayed exacerbated schizophrenic-like behavior from the low-dose PCP treatment, which may explain the milder effects of PCP on sociability scores. Mice often followed and circled close to each other, while sniffing each other briefly but more continuously than WT mice. Beyond susceptibility, the P2rx7^tg/+^ mice experience a strongly accentuated stereotypical behavior which correlates with values of concentration of strongly stained c-Fos neurons in the same area where were reduced in P2rx7^−/−^, the layer V of IL and PL areas. The higher PCP sensitivity in terms of behavior and medial prefrontal neuronal hyperactivation adds credibility and relevance to the model, as patients with schizophrenia have an increased response to arylcyclohexylamines anesthetics relative to dopamine-related excitant drugs (Lahti et al., 2001). Interestingly, the lower dopamine uptake of P2rx7^tg/+^ animals suggests that the overexpressing strain has prefrontal hyperdopaminergia, which might also account for the exacerbated PCP effect. The P2X7-EGFP, expressed by heterozygous P2rx7^tg/+^ mice, was localized in the layer V mPFC microglia but was expressed by other cells throughout the mPFC.

To summarize our findings, P2rx7^−/−^ animals might display small cumulative effects pointing to a hypo-excitability mPFC circuit and tempered psychotomimetic PCP effect, namely:

- The lower dopaminergic tone in the PFC.
- PL and IL layer V enhanced microglial branches/hyperactive neuron interactions.
- PL and IL layer V neurons with faster post-action potential firing polarization and lower initial action potential frequency.

While symptoms related to psychotic schizophrenic episodes, which are mimicked by the acute PCP effect, can be alleviated by the available antipsychotic drugs, there is an unmet clinical need concerning cognitive deficits (Tripathi et al., 2018). We examined the working memory of P2rx7^−/−^ mice using an established subchronic PCP treatment that compromises memory performance (Zain et al., 2018). Along with the treatment, PCP tended to induce a smaller acute hyperlocomotion in knock-out animals, without compromising the behavior in the short-term washout. After the subchronic treatment withdrawal period of 3 days, the animals were tested for the spontaneous alterations test in a Y-maze. The lower locomotion displayed by P2rx7^−/−^ animals in the Y-maze was contextual to this test, as we could not rule out genotype locomotion-related differences in tests before and after the spontaneous alternation test. As mentioned, we did not take into consideration the repeated entries in the same arm as “valid alternations”. Therefore, a different velocity between genotypes may reflect different behavioral explorative strategies. However, these did not influence the total number of different arm explorations, and thus alternation, during the test. Subchronic PCP treatment significantly impaired the working memory performance in wild-type mice concerning the P2rx7^+/+^ vehicle group and the P2rx7^−/−^ PCP treated group. The following analysis of the prefrontal cytokine levels did not reveal any PCP-related effects on the brain inflammatory profile. These data confirm that the noncompetitive antagonism of NMDA receptors directly modulates neuronal activity and does not trigger a robust neuroinflammatory phenomenon. Interestingly, the prefrontal level of fractalkine, or CX3CL1, was strongly upregulated in both genotypes subjected to PCP treatment. Fractalkine is a pivotal trophic factor of the brain and is strongly involved in microglial physiology, with many implications (Arnoux and Audinat, 2015). Since P2X7R also has a trophic effect on microglia (Bianco et al., 2006; Monif et al., 2016), we cannot exclude the possibility that fractalkine upregulation could play a compensatory role in P2rx7^−/−^ animals.

In addition to the mechanistic explanations detailed above, we cannot exclude different pharmacokinetics of PCP in the different genotypes. In rats, PCP has a fast brain uptake and hours-timescale washout (Kalinichev et al., 2008), and subchronic PCP is cumulated in plasma and tissues (Balla et al., 2003). Arime and Akiyama reported that when rodents were involved in working memory tasks, there was increased recruitment of neurons at the level of the prelimbic layer II/III, which correlated with poorer performance (Arime and Akiyama, 2017).

Acute PCP treatment is also known to trigger a stress response, affecting the hypothalamus-pituitary-adrenal axis by stimulating pituitary ACTH release and consequently increasing plasma corticosterone levels (Pechnick et al., 1990, 2006). After PCP subchronic treatment, induced stress triggers greater stress responses in terms of behavior and plasma levels of ACTH (Tejedor-Real et al., 2007). Interestingly, our group previously published evidence that P2rx7^−/−^ animals exhibit an anti-depressed profile, since they respond better to a broad spectrum of stressors (physical restraint, amphetamine, and lipopolysaccharide treatments) by reducing centrally dependent HPA axis activation (Csölle et al., 2013). This, along with other evidence, points to a hypothetical cascade of neuroinflammatory-related events that would explain the protective role of P2X7R against repetitive stressful events (Illes et al., 2020). Also, our previous study (Koványi et al., 2016) pointed to a set of subtle expression changes in several genes implicated in SCZ in a small dose acute PCP model, which was partially reversed in P2rx7^−/−^ animals.

Collectively, our findings support the notion that P2X7R exerts multiple roles, depending on the context and the cell type. It seems reasonable to assume that short-term activation of P2X7R could impact the functionality of neuronal networks in the long term, such as gene expression regulation, behavior, and memory formation. The P2X7-driven action identified by the current study, such as increased layer-specific neuronal activation and intrinsic excitability of neurons in the mPFC and preferential interaction of microglia with hyperactive neurons, might be responsible for the alterations detected in PCP-induced behavior. However, the mechanism by which P2X7R is endogenously activated under these conditions needs to be further examined.

## Conclusion

These results confirm the role of P2X7R in SCZ-like behaviors in an animal model, which we propose as a potential therapeutic target. A decrease of neuronal activation in a restricted area of the mPFC in response to PCP treatment in mice genetically deficient for P2rx7 was observed. Specifically, P2X7R gene deletion elicited a lower number of strongly activated neurons in the mPFC specific to the V layer of the prelimbic and infralimbic areas. Opposite results were found testing a P2X7R overexpressing line, which is more susceptible to PCP in terms of behavior and prefrontal specific circuits involvement. This finding identifies a potential primary site of action, whereby P2rx7 might affect PCP-induced behavior. Moreover, we observed a change in microglial morphology in the same area regarding the contact sites of microglia over the neuronal somata. This raises the possibility that P2X7 regulates microglia-neuron interaction and thereby affects animal behavior.

## Data Availability Statement

The raw data supporting the conclusions of this article will be made available by the authors, without undue reservation.

## Ethics Statement

The animal study was reviewed and approved by Local Animal Care Committee of the Institute of Experimental Medicine (Budapest, Hungary, ref. No. PEI/001/778-6/2015). International Council for the Laboratory Animal Science at the University of the Basque Country UPV/EHU (CEEA 290/2015).

## Author Contributions

SC, BS, ESV, and JT designed the research. SC, AI, PT, and PM-A performed the research and analyzed the data. AN and SM provided transgenic mouse models. BS, ESV, and JT supervised the study. SC wrote the article with input from all authors. All authors contributed to the article and approved the submitted version.

## Conflict of Interest

The authors declare that the research was conducted in the absence of any commercial or financial relationships that could be construed as a potential conflict of interest.
